# Spatio-temporal patterns and trends of the air pollution integrating MERRA-2 and in situ air quality data over Egypt (2013–2021)

**DOI:** 10.1007/s11869-023-01357-6

**Published:** 2023-05-19

**Authors:** Alaa A. Masoud

**Affiliations:** Remote Sensing Laboratory, Geology Department, Faculty of Science, Tanta University, Tanta, 31527 Egypt

**Keywords:** Seasonal Mann–Kendall, Air quality, Annual change rate, Egypt

## Abstract

For best-informed decision-making to improve climate change adaptation and reduce present and future air pollution health hazards, it is essential to identify major trends in spatiotemporal air quality patterns of common air contaminants. This study examined the patterns and trends of SO_2_, NO_2_, CO, O_3_, and particulate matter (PM) air pollutants over 91 monitoring stations in Egypt during 93 months in the August (2013)–April (2021) period. In situ data with their monthly, seasonal, and yearly spatial trends are defined and used to validate the counterpart satellite reanalysis MERRA-2 data. The Mann–Kendall test characterized the seasonal monotonic trends and their Sen’s slope, and annual change rate for both data series. Regression analysis of MERRA-2 against in situ concentrations of SO_2_ and PM_10_ revealed underestimation with RMSE values of 13.38 g m^−3^ and 69.46 g m^−3^, respectively. Local plumes with variable magnitudes characterized distinct industrial places clarified by patterns of in situ pollutants. As a result of the COVID-19 lockdown, the in situ air pollutants showed a considerable regional decline in the yearly average in 2020 compared to the years before. The in situ air pollutants showed annual trends far more significant than those seen in the MERRA-2 data. The shortcomings of the few and spatiotemporal discontinuities of the in situ contaminants are addressed by MERRA-2 air quality products. The in situ data made trends and magnitudes clear that were hidden in their MERRA-2 counterparts. The results clarified air pollution patterns, trends, and spatial variability over Egypt that are essential for climate risk management and for reducing environmental/health concerns.

**Supplementary Information:**

The online version contains supplementary material available at 10.1007/s11869-023-01357-6.

## Introduction

The Earth’s climate changes pose a severe threat to human health (Cianconi et al. 2020; Zheng et al. 2021) caused by poor air quality (Ding et al. 2017; Hao et al. 2017), and have become a focus for global environmental policy-making (Burnett et al. 2018). The increasing numbers of premature deaths and disability-adjusted life years (DALY) have been linked to ambient air pollution (Tomczak et al. 2016), which was responsible for 5.9% of total DALY and 8.8% of deaths from all causes worldwide in 2017 (GHDx 2019) as the fourth most significant factor of raising global mortality risk in the Global Burden of Disease (GBD) study 2015 (Forouzanfar et al. 2016), outdoor air pollution is forecasted to contribute to double the global mortality burden by 2050 (Lelieveld et al. 2015). With the rapid increase in population and overexploitation of natural resources, climate changes also adversely affect ecosystem services (Stuch et al. 2020) and socio-economic development as well (Ma and Zhang 2014).

Over the past several decades, due to the quick pace of industrialization and urbanization, and a lack of effective comprehensive pollutant abatement, many world countries are still facing many challenges from air pollution. Among the key determinants of current and future climate change impacts on air quality are gaseous emissions and particulate matter variability, and shifts in the magnitude of these variables are associated mostly with global warming. Human-induced warming reached approximately 1 °C above pre-industrial levels in 2017 (IPCC 2014), increasing by 0.2 °C per decade. In large parts of Africa, the Earth’s surface temperature has risen by at least 0.5 °C in the past 50–100 years. Warming could become more severe if the net-zero emission ambitions of COP26 are not met. WHO air quality database reveals that 97% of affected cities are in developing low- and middle-income countries with more than 100,000 inhabitants (WHO, 2018b). Ambient air pollution is due to high concentrations of airborne particulate matter (PM), ozone (O_3_), nitrogen dioxide (NO_2_), carbon monoxide (CO), and sulfur dioxide (SO_2_), which have adverse health effects (Mannucci and Franchini 2017).

A better understanding of the spatio-temporal variability in surface air pollutants concentration is induced by emission characteristics, meteorology, topography, density and distribution of monitoring stations, analytical methods, instrument types and quality, and expertise in measuring and data analysis. Commonly, most monitoring stations are installed in densely populated urban areas, the distribution of which is scarce and dispersed in less-accessible areas. This hinders adequate spatial and temporal coverage for consistent air quality data. With the rapid advancement in space technology, satellite-based aerosol optical depth (AOD) products are now being used to overcome the shortcomings of bottom-up inventory in retrieving variations in chemical species composition (Levelt et al. 2018).

The reproduction capability of aerosol spatial distribution (Shin et al. 2019) has been improved by integrating models from the various ground- and space-based remote sensing platforms, such as the Modern-Era Retrospective Analysis for Research and Application, version 2 (MERRA-2) model. MERRA-2 is the most up-to-date modern satellite era (1980 onward) atmospheric reanalysis from the NASA Global Modeling and Assimilation Office (GMAO; Gelaro et al. 2017) to include additional observations and numerous improvements to the Goddard Earth Observing System, version 5 (GEOS-5), Earth system model (Molod et al. 2015), representation of the hydrologic cycle (Takacs et al. 2015), the stratosphere, ozone, and cryospheric processes (Bosilovich et al. 2016).

Previous studies had reported that the MERRA-2 dataset captured the temporal and spatial sequential changes in AOD very well, except for heavy pollution (Buchard, et al. 2017; Sun, et al. 2019), when compared with other satellites like the Medium Resolution Imaging Spectroradiometer-MODIS (Wei 2021). An increasing number of studies have combined pollutant ground measurements and MERRA-2 reanalysis data to evaluate MERRA-2 for black carbon (Qin et al. 2019; Xu et al. 2020; Sitnov et al. 2020), carbon monoxide (García-Franco 2019), PM2.5 (Song et al. 2018), and for air quality forecasting (Mukkavilli et al. 2019), in many world regions. Still, the majority of satellite applications have not been validated in developing countries, typically of high pollution levels and distinctive emission source profiles (Liu 2013; Cabaneros et al. 2019). This has historically been difficult worldwide, as in Egypt, requiring long-term and high-quality dense in situ observations of wide geospatial coverage which has been recently increasingly available in Egypt.

Based on Global Burden of Disease 2017 results, Egypt’s population is projected to reach about 200 million by 2100 (Vollset et al. 2020), which, along with continued climate changes, will exacerbate the problem of air pollution, posing challenges to many aspects of development. World Health Organization (WHO 2018a, b) stated that over 43,000 Egyptians died in 2012 from air pollution-related diseases comprising acute lower respiratory, chronic obstructive pulmonary disease, stroke, ischemic heart disease (IHD), and lung cancer. According to the report, 22,327 Egyptians died of IHD while 2079 lost their lives to lung cancer. According to the World Bank (2019), relying on 2016–2017 data, air pollution in the Greater Cairo Metropolitan Area (GCMA) alone costs EGP 47 bn in 2017, which was equivalent to 1.35% of Egypt’s GDP where the population (millions) exposed to PM_2.5_ increased by 45% from 11.9 in 1999 to 17.3 in 2017, with annual deaths raised by 34% from 9400 to 12,569, respectively. Air pollution shortens the life expectancy of Egyptians by two years on average due to morbidity or disability (Apte et al. 2018). Air pollution was the cause of 90,559 premature deaths in Egypt in 2019 (UNEP 2022), and more than 12% of all deaths in 2017 (Institute for Health Metrics and Evaluation 2019). According to the WHO database, air pollution-related illnesses responsible for premature mortality in Egypt in 2016 included heart disease (57.9%), stroke (17.7%), and pulmonary and lower respiratory diseases and cancer (24.4%) (WHO 2018). Noncommunicable diseases are the number one cause of death in Egypt, responsible for 82% of deaths and 67% of premature deaths (WHO 2022).

Egypt is taking dire steps to mitigate the impact both of air pollution and of climate change. Egypt’s Vision 2030 has set a target of reducing PM_10_ small particulate matter concentrations by 50% by 2030. Furthermore, it has formulated a greenhouse gas (GHG) emissions reduction action plan (World Bank 2021), the Intended Nationally Determined Contribution (INDC), as part of its commitment to the Paris Climate Agreement, and the UN’s Framework Convention on the 2021 Climate Change Conference of Parties (COP21).

It is challenging to implement effective air pollution management strategies based on fact-informed decisions since patterns and trends of elevated levels of air pollutants from poorly managed sources have not yet been quantified in Egypt. To create targeted and efficient actions to improve air quality, a thorough emission inventory focused to comprehending spatiotemporal differences in air pollution is essential. The research examines the variation in atmospheric levels of MERRA-2 air pollutants SO_2_, CO, NO_2_, O_3_, PM_1_, PM_2.5_, and PM_10_, as well as the ground-truth in situ data of SO_2_, NO_2_, and PM_10_ and comparing them with MERRA-2 data for 93 months (August 2013–April 2021) at 91 monitoring sites over Egypt. Finding the most polluted locations, describing those with statistically significant trends, and revealing their annual change rates are the overall main goals. To the best of the authors’ knowledge, this study is the first to use reanalysis data—specifically, aerosol and meteorology products from the MERRA-2 satellite—to characterize patterns and trends in air pollution in Egypt. This will help the community be more prepared and assist the best-informed decision-making about plans for adaptation and mitigation to lessen the effects of air pollution on future health and the environment.

## Data and methods

**Fig. 1:**
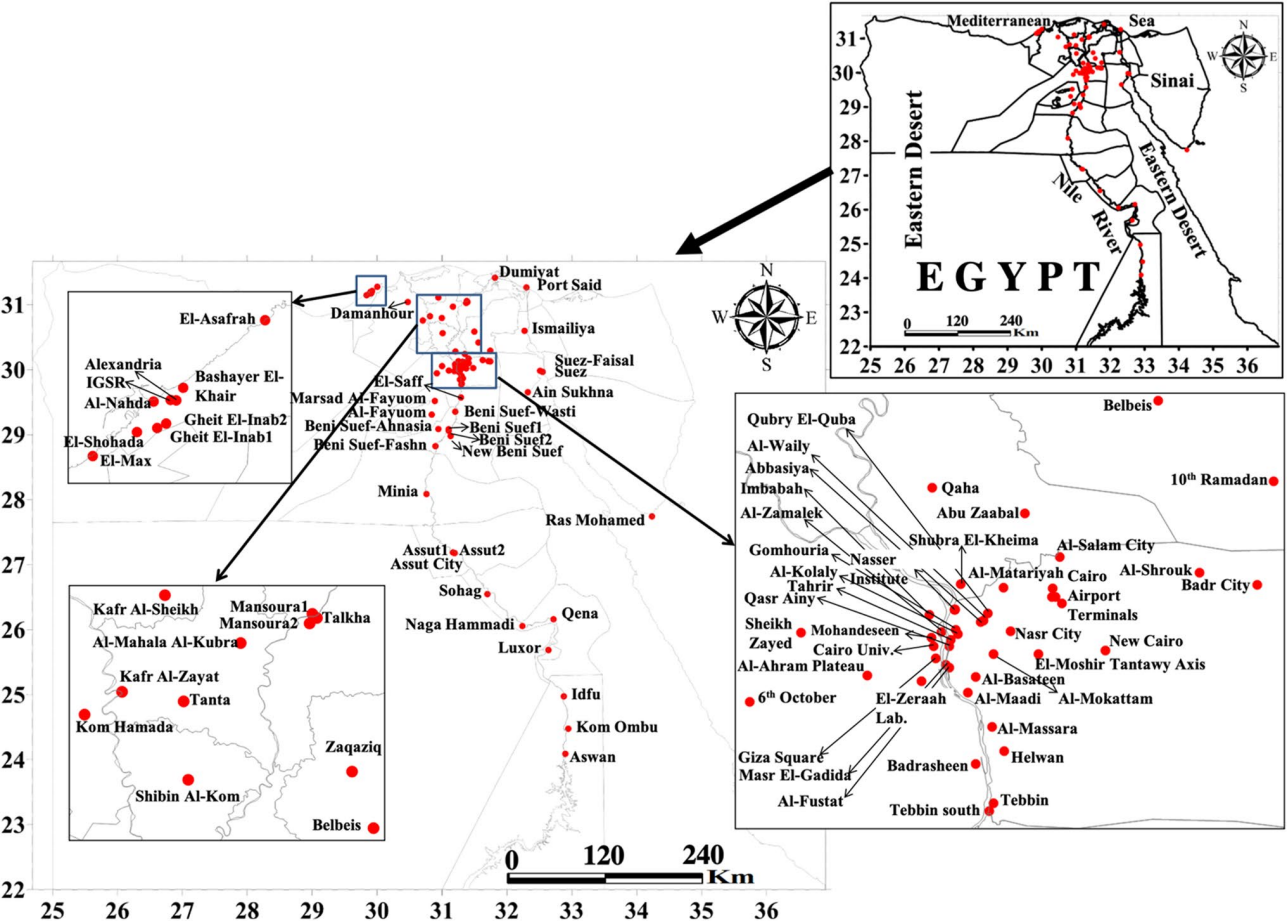
Location map of the study area with air quality monitoring stations marked in red-filled circles

In the present research, air pollution patterns and trends are evaluated for 93 monthly averages of air quality data estimated from the satellite reanalysis data of MERRA-2 products and validated using in situ ground truth measurements. MERRA-2 air quality data included SO_2_, CO, and O_3_, and particulate matter at varying micron sizes of 1 (PM_1_), 2.5 (PM_2.5_), and 10 (PM_10_). The recently made available long-term air quality data from the monitoring network of 91 stations across Egypt, denser in the populous GCMA and the Nile Delta regions and sparse everywhere, provided the base for comprehensively assessing the long-term trends of air quality at the nation scale (Fig. 1). Forty-two stations are concentrated in the GCMA from Qaha in the north to El-Saff in the south and from Badr City in the east to 6^th^ October in the west. Nineteen stations are distributed in areas close to the Nile riverbanks of the Upper Egypt governorates from Al-Fayoum in the north to Aswan in the south. Nine stations are located in Alexandria. Twelve stations are distributed in the Nile Delta. Five stations are located in the Suez Canal economic zone (3 in Suez, Ismailiya, and Port-Said). In addition to 10^th^ of Ramadan, Belbeis, and Zaqaziq in Sharqiya governorate, and Ras Mohamed in South Sinai.

Air quality satellite data over Egypt is based on the latest atmospheric reanalysis release in 2017 by the NASA GMAO (Global Modeling and Assimilation Office), that is the MERRA-2 (Modern-Era Retrospective analysis for Research and Applications, Version 2) (Buchard et al. 2017; Randles et al. 2017). Data has been downloaded in the form of time-averaged 2-dimensional monthly mean data collection (tavgM_2d_aer_Nx) covering Egypt for 93 months (August 2013–April 2021). Simulated with 72 vertical layers from the surface to higher than 80 km using the GEOS-5 (GMAO Earth system model version 5) model radiatively coupled to the GOCART (Goddard Chemistry Aerosol Radiation and Transport) model (Chin et al. 2002; Colarco et al. 2010), that considers the sources, sinks, and chemistry of 15 externally mixed aerosol species as column mass density of aerosol components (dust in 5 size bins, sea salt in 5 size bins, hydrophilic and hydrophobic organic carbon (OC) and black carbon (BC), and sulfate), surface mass concentration of aerosol components, and total extinction (and scattering) aerosol optical thickness (AOT) at 550 nm. These data are used to generate MERRA-2 aerosol gridded products such as that of SO_2_, BC, OC, tropospheric ozone, dust, sea salt, and sulfate at a spatial resolution of 0.625° (longitude) × 0.5° (latitude). The total PM_1_, PM_2.5_, and PM_10_ are derived, with the variance of certain parameters, from the formula described in the FAQs under the Documentation tab of MERRA-2 Specs.

For evaluating the satellite data, in situ data based on 93 months of paper-based histograms with monthly records noted on the top of the columns secured by the Egyptian Environmental Affairs Agency (EEAA 2022) is used. Excel worksheets are prepared with records that are manually transferred to the sheets for the three pollutants: SO_2_, NO_2_, and PM_10_. Available data varied in length and continuity.

For detecting the trends, the Seasonal Mann–Kendall (SMK) nonparametric test is then performed on MERRA-2 and the ground truth in situ data with a 95% confidence level (alpha = 0.05). For each variable across all stations, the Sen slope reflecting the annual change rate is calculated, along with the SMK parameters of the score *S*, *S* Variance, and Tau. Where the slope is the same but with different *S* Variance and tau values, serial dependency and independence—whether assuming serial correlations among adjacent consecutive periods (months) exist or not—are examined. SMK adopts a consistent slope direction during the whole observation period.

In order to quantify the anthropogenic loads on air pollutants, we used the Gridded Population of the World collection version 4 (GPWv4.11) at roughly 1 km, 5 km, 30 km, 55 km, and 110 km (CIESIN 2018). The population densities at various scales are extracted using coordinate points from monitoring stations. The averages of the monitoring period for MERRA-2 and in situ air contaminants, regressed against population densities, are examined for Pearson’s correlation and determination coefficients.

Furthermore, estimation of the particulate matter, statistical analyses performed, seasonal trends and heterogeneity tests carried out are all described in 4.

### Particulate matter estimation

The concentration of PM_x_ at varying sizes can be computed using fields from the tavgM_2d_aer_Nx data collection applying Eqs. (1–3):

1 $${\mathrm{PM}}_{1}=\left(1.375*\mathrm{SO}4+\mathrm{BC}+\mathrm{OC}+0.7*{\mathrm{Dust}}_{1}+{\mathrm{SS}}_{1+2}\right)*\mathrm{AIRDENS}$$

2 $${\mathrm{PM}}_{2.5}={\mathrm{Dust}}_{2.5}+\mathrm{OC}+\mathrm{BC}+{\mathrm{SS}}_{2.5+}1.375*{\mathrm{SO}}_{4}$$

3 $${\mathrm{PM}}_{10}=\left(1.375*{\mathrm{SO}}_{4}+\mathrm{BC}+\mathrm{OC}+{\mathrm{Dust}}_{\left(1+2+3\right)}+0.74*{\mathrm{Dust}}_{4}+{\mathrm{SS}}_{\left(1+2+3+4\right)}\right)*\mathrm{AIRDENS}$$

The subscripts of the dust (Dust) and sea salt (SS) are the pins summed to estimate both parameters. AIRDENS is the air density, whereas, Dust_2.5_, SS_2.5_, BC, OC, and SO_4_ are the GOCART concentrations of dust, sea salt, black carbon (phobic and philic), organic carbon (phobic and philic), and sulfate in particles with a diameter smaller than 2.5 μm, respectively. Sulfate requires a multiplication factor since the species tracer in MERRA-2 is the sulfate ion and does not include nitrate aerosol. The values were very small and therefore converted to micrograms per cubic meter. Tropospheric ozone unit is in Dobson.

### Statistical methods

The data series is subjected to statistical analysis, and estimations of descriptive summary statistics—including minimum and maximum values for monthly, seasonal, and yearly means, standard deviation, and percentiles—as well as other statistical parameters are made. The cross-validation of the in situ and MERRA-2 time-series data is measured using the root mean squared error (RMSE). For statistical data analysis, XLSTAT, an Excel add-in, is employed along with SMK and a number of heterogeneity tests. ArcGIS version 9.3 was used to analyze the spatial distribution of air pollution concentrations, together with their statistics and rate of change. Spherical models were frequently used, and krigging variogram modelling was evaluated. In order to obtain the seamless regional approximation with the lowest error statistics, maps are cross-validated against the original data.

### Seasonal trend and heterogeneity tests

Statistical monotonic trend tests in seasonal (e.g., monthly) environmental and climate time series data are commonly confounded by non-normal data, missing values, seasonality, censoring (detection limits), and serial dependence (Sicard et al. 2013). An extension of the Mann–Kendall test for trend (designed for such data) implemented in XLSTAT is adopted and applied in this research. Because the test is based entirely on ranks, it is robust against non-normality and censoring. Seasonality and missing values present no theoretical or computational obstacles to its application. MERRA-2 and in situ ground-truth data were analyzed using the non-parametric SMK test and Sen’s slope method to statistically assess the linear upward or downward trend of the variable of interest over time and the magnitude of change (Mann 1945; Sen 1968; Kendall 1975; Gilbert 1987). The traditional MK test is described first followed by the modifications for seasonality. MK assumes a null hypothesis (*H*_o_) of no monotonic trend in the data series while its alternative hypothesis (*H*_a_) assumes that there is a presence of a monotonic trend in the time-series data.

## Mann–Kendall trend test

In the traditional MK analysis, the number of sequential values in the studied data series is denoted by *n*. If *n* is 9 or less, the absolute value of *S* is compared directly to the theoretical distribution of *S* derived by Mann and Kendall (Gilbert 1987). The MK test statistic *S* is calculated using Eqs. (4) and (5).

4 $$S={\sum }_{k=1}^{n-1}{\sum }_{j=k+1}^{n}sgn\left({x}_{j}- {x}_{k}\right)$$

where,

5 $$sgn\left({x}_{j}- {x}_{k}\right)= \left\{\begin{array}{c}1 if {x}_{j}- {x}_{k} >0\\ 0 if {x}_{j}- {x}_{k} =0\\ -1 if {x}_{j}- {x}_{k} <0\end{array}\right.$$

*x*_*j*_ and *x*_*k*_ are the sequential monthly data values. When *S* bears a positive value, it indicates an upward or increasing trend and if the value is negative, it indicates a downward trend or decreasing trend. If *n* is at least 10 or more, the test follows a normal distribution; and hence, a normal approximation test is used with expectation (*E*) and variance of *S* as *VAR*(*S*) using Eq. (6):

6 $$VAR \left(S\right)= \frac{1}{18} \left[n \left(n-1\right)\left(2n+5\right)- {\sum }_{p=1}^{q}{t}_{p}({t}_{p}-1)(2{t}_{p}+5)\right]$$

Here, *q* is the number of tied groups and *t*_*p*_ is the number of data points in the *p*^th^ tied group in the dataset. The standardized test statistic (*Z*) is calculated as Eq. (7):

7 $$Z= \left\{\begin{array}{c}\frac{S-1}{\sqrt{VAR(S)}} if S>0\\ 0 if S=0\\ \frac{S-1}{\sqrt{VAR(S)}} if S<0\end{array}\right.$$

where, the value of *Z* is the MK test statistic which follows a standard normal distribution with the mean being 0 and variance being 1. In this study, confidence intervals of 95% (*p* < 0.05) were taken to classify the significance of positive and negative trends. Furthermore, the Sen slope estimator of the linear trend has been estimated using the Theil–Sen estimator (Sen 1968). The slope (*Q*) estimates of *N* pairs of data are first computed by Eq. (8):

8 $${Q}_{i}= \frac{{x}_{j}{- x}_{k}}{j-k};For i=1, 2, 3, \dots \dots .N.$$

where *x*_*j*_ and *x*_*k*_ are data values at times *j* and *k* (*j* > *k*) respectively. The median of these *N* values of *Q* is Sen’s estimator of the slope.

## Seasonal Mann–Kendall and homogeneity tests

The SMK (Hirsch et al. 1982), modified from Kendall’s test, is for the trend that allows for seasonality in observations collected over time, which is appropriate for trend testing in each season when the trend is always in the same direction across all seasons.

The MK statistic for the *g*^th^ season is calculated using Eq. (9):

9 $${S}_{g}={\sum }_{k=1}^{n-1}{\sum }_{j=k+1}^{n}sgn\left({x}_{jg}- {x}_{kg}\right), g=\mathrm{1,2},\dots ,m$$

The SMK statistic, *Ŝ*, for the entire season series (12 months, in this study) is calculated using Eq. (10) as follows:

10 $$\widehat{S}=\sum_{g=1}^{m}{S}_{g}$$

For further information, the reader is referred to Hipel and McLoed (1994, p. 866–869) and Hirsch et al. (1982). More details for the seasonal Mann–Kendall test are available at https://cran.r-project.org/web/packages/trend/vignettes/trend.pdf.

The assessment of climate-related data reliability is mainly done by performing homogeneity tests (Hänsel et al. 2016) which detect and define inflection point (month in a year) abrupt changes that mostly mark climatic or anthropogenic extremes related to changes in instruments, observation practices, station geographical location, calculations codes and units, and land use/cover for the trend in any direction in any season to help identify non-climatic environmental factors contributing to spatio-temporal variability (Peterson et al. 1998). Homogeneity tests implemented in XLSTAT software (van Belle and Hughes 1984) were performed using three test methods (i.e., Pettitt’s, SNHT, and Buishand's test) at monthly timescales. In this study, the null hypothesis was accepted and the data were considered homogeneous when the computed *p*-value for each test was greater than the significance level (0.05). For the inhomogeneous series, the inflection points are identified.

## Results

### MERRA-2 air pollution patterns of trends

**Fig. 2:**
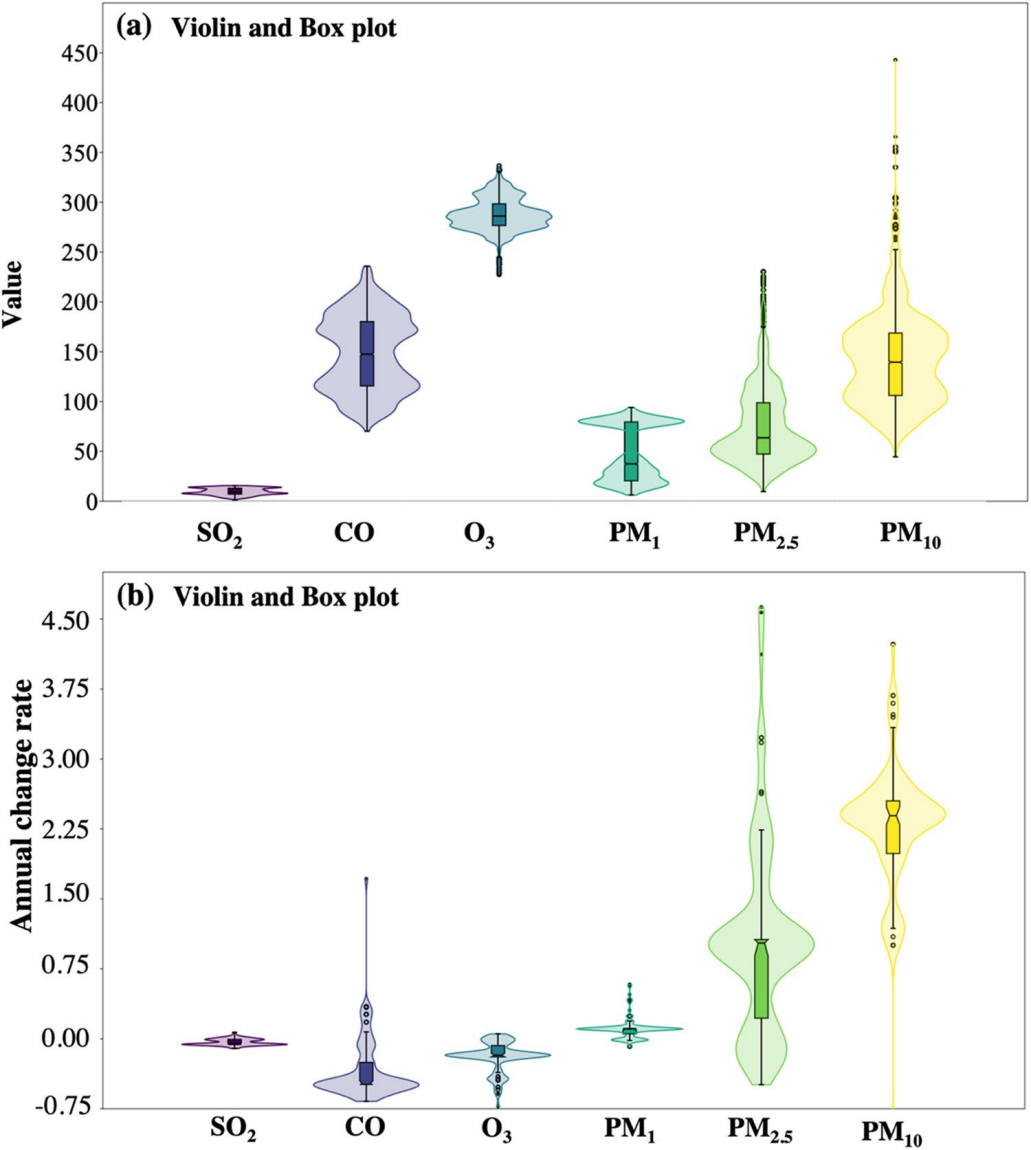
Violin and Box plots of the MERRA-2 derived air pollutants showing **a** concentration, and **b** annual change rate

Violin and Box plots along with descriptive statistics of the air pollutants contents and magnitude of monotonic trends estimated from MERRA-2 are shown on Fig. 2 and Table 1, respectively. The amount of pollutants showed wide variations. SO_2_ has a range of of 44–15.6 μg m^−3^and an average of 9.6 μg m^−3^. CO averaged 148 μg m^−3^ in a range level of 70–236 μg m^−3^. Tropospheric ozone varied between 227 and 336 Du, with an average value of 288 Du. PM_10_ leads the content of the PM with an average of 142 μg m^−3^, followed by 75 μg m^−3^ PM_2.5_, and then 47 μg m^−3^ PM_1_. Their range levels also varied with 44–443 μg m^−3^ PM_10_, 9.7–230 μg m^−3^ PM_2.5_, and 6.2–94 μg m^−3^ PM_1_. PM_10_ and PM_2.5_ contents and trends are highly influenced by outliers (Fig. 2).

**Table 1:** Descriptive statistics of MERRA-2 air pollutants

(*n* = 8463)	SO_2_	CO	O_3_	PM_1_	PM_2.5_	PM_10_
Min	1.44	70	227	6.2	9.7	44
Max	15.6	236	336	94	230	443
Mean	9.6	148	288	47	75	142
Stand. dev	3.4	37.9	15.7	28.1	37.0	45.1
25 percentile	7.2	115.8	276.7	20.5	47.3	106.1
75 percentile	12.9	180.2	298.4	79.5	98.7	168.7
Coeff. var	35.6	25.5	5.4	59.1	49.4	31.8

Correlation coefficients among the studied MERRA-2 air pollutants (Table 2) indicated a significant strong relationship of the PM_1_ (*r* = 0.83), CO (*r* = 0.82), and PM_10_ (*r* = 0.47) with the SO_2_ concentration. CO is greatly influenced by PM_1_ (*r* = 0.82) and PM_10_ (*r* = 0.57). These results confirm that SO_2_ and CO coexist (*r* = 0.82), as well as PM_1_ and PM_10_ (*r* = 0.74). Also, PM_1_ and PM_10_ constitute the major part of SO_2_ and CO. PM_2.5_ contributes largely to the ozone content (*r* = 0.42). And so, aerosol chemical composition is heavily affected by dust winds from deserts, with some contribution of local traffic and industries, a result which has been proven by Kchih et al. (2015).

**Table 2:** Pearson’s correlation coefficients of the MERRA-2 air pollutants

b	SO_2_	CO	O_3_	PM_1_	PM_2.5_	PM_10_
SO_2_	1					
CO	0.82	1				
O_3_	0.07	0.02	1			
PM_1_	0.83	0.82	0.13	1		
PM_2.5_	− 0.11	− 0.11	0.42	0.01	1	
PM_10_	0.47	0.57	0.28	0.74	0.24	1

**Fig. 3:**
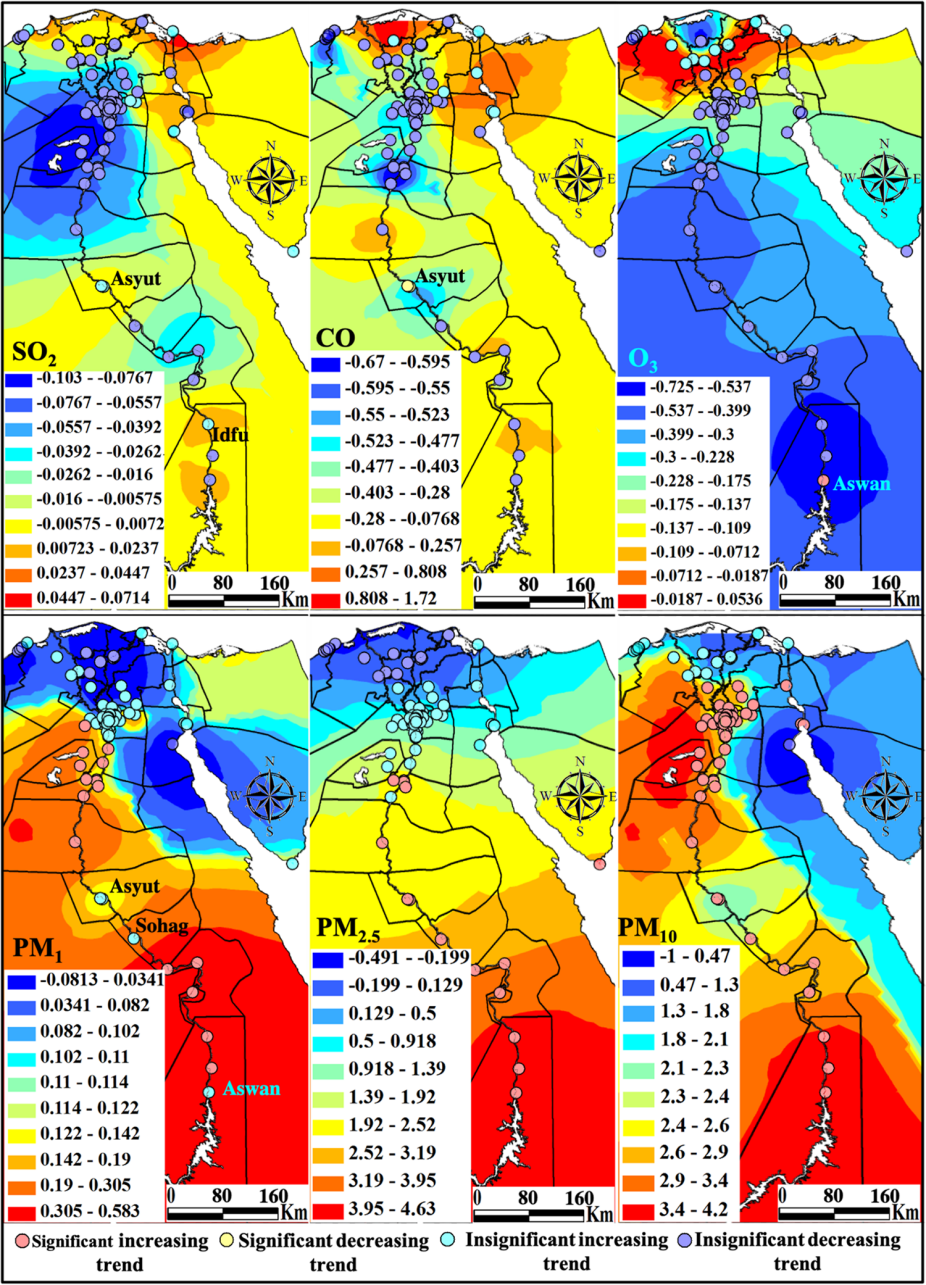
Maps showing the annual change rate of the MERRA-2 air pollutants, significant or insignificant, and increasing or decreasing trends

The largest annual average rate of the 91 monitoring stations over Egypt marked the PM_10_ (2.28 μg m^−3^), followed by PM_2.5_ (1.07 μg m^−3^), PM_1_ (0.11 μg m^−3^), SO_2_ (− 0.04 μg m^−3^), O_3_ (− 0.20 Dobson), and CO (− 0.34 μg m^−3^), in decreasing order, as listed in Table 3. Patterns of monotonic trend magnitudes are spatially displayed on Fig. 3. All stations showed insignificant increasing or decreasing SO_2_ trends. Two plume areas of SO_2_ do exist in the Suez Canal economic zone and in the Aswan-Idfu area confirming the same result obtained from the in situ data explained later with Ain Sukhna and Port Said showed increasing trends but of less significance compared to that of the in situ data. Suez and Ismailiya experienced insignificant decreasing trends. Idfu and Aswan clarified insignificant increasing and decreasing trends, respectively. The pattern of CO trends is similar to that of SO_2_ as they are mutually co-emitted from similar pollution sources. Increasing insignificant trends mark the Suez Canal Economic Zone (SCZone) stations; Suez, Ismailiya, and Port Said. A local plume of increasing insignificant CO trends at a rate in the level range of 0.80–1.72 μg.m^−3^ persists over Kafr Al-Sheikh governorate from 2013 until 2021. Observations of total ozone in Egypt revealed insignificant declining trends, however, with an exceptional zone of increase near 0.05 DU from Port Said, Dumiyat, middle Delta stations, Damanhour, and El-Asafrah (Alexandria).

**Table 3:** Descriptive statistics of the MERRA-2 air pollutants annual change rate at all stations

(*n* = 91)	Min	Max	Mean	Std. dev
SO_2_	− 0.10	0.02	− 0.04	0.03
CO	− 0.62	1.72	− 0.34	0.34
O_3_	− 0.73	0.05	− 0.20	0.16
PM_1_	− 0.08	0.58	0.11	0.12
PM_2.5_	− 0.21	4.63	1.07	1.05
PM_10_	− 1.01	4.23	2.28	0.68

In general, increasing particulate matter (PM_1_, PM_2.5_, and PM_10_) trend magnitudes propagate gradually southward to reach the maximum annual rate over Aswan at about 4 μg m^−3^. This is the result that has been achieved in southern European countries (Spain, Portugal, and Italy) affected by PM originating from the African Sahara Desert. These trends persist from south to east Delta stations (Shibin Al-Kom and Zaqaziq, and Ismailiya) and continue southwards over Upper Egypt. PM10 showed the most widespread significant increasing trends, followed by PM_1_, and PM_2.5_ came last. Although middle and north Deltas varied in their significance, PM_10_ increased, and PM_2.5_ decreased; for PM_1_, increasing trends occurred further from deserts in the eastern and western delta fringes, while decreasing trends were observed at the middle Delta monitoring stations, located amidst agricultural areas.

Pearson’s correlation analysis at a significance level of 95% that is shown on Table 4 indicated mutual trend coexistence of SO_2_ and CO (*r* = 0.48) greenhouse gas emissions. Particulate matters of PM_1_ strongly positively correlate with PM_2.5_ (*r* = 0.91) and PM_10_ (*r* = 0.61), while PM_2.5_ form a large part of the PM_10_ (*r* = 0.51). Tropospheric ozone showed a strong negative correlation with the particulate matters of PM_2.5_ (*r* =  − 0.83), PM_1_ (*r* =  − 0.71), and PM_10_ (*r* =  − 0.37), in decreasing order.

**Table 4:** Pearson’s correlation coefficients of MERRA-2 air pollutants annual change rate

(*n* = 91)	SO_2_	CO	O_3_	PM_1_	PM_2.5_	PM_10_
SO_2_	1					
CO	0.48	1				
O_3_	0.02	− 0.24	1			
PM_1_	0.00	0.13	− 0.71	1		
PM_2.5_	− 0.06	− 0.04	− 0.83	0.91	1	
PM_10_	− 0.38	− 0.28	− 0.37	0.61	0.51	1

Bold values are significant at 95% level

### In situ air quality spatio-temporal variations

#### Statistical variation of air pollutants

The number of measurements and length of continued monitoring varied largely among in situ air pollutants at all stations, with SO_2_ being the longest and largest in the number of records (*n* = 2834), followed by PM_10_ (*n* = 2577), and then NO_2_ (*n* = 1973). Descriptive summary statistics are shown in Table 5. SO_2_ clarified an average of 15.2 μg m^−3^ with level range of 0–187 μg m^−3^. NO_2_ averaged 30.6 μg m^−3^ in the range level of 1–161 μg m^−3^. PM_10_ showed an average of 137.7 μg m^−3^ and a range of 12–437 μg m^−3^. Spatio-temporal variations of the studied air pollutants are described in the following sections.

**Table 5:** Descriptive statistics of the in situ air pollutants

Variable	No. of records	Minimum	Maximum	Mean	Std. deviation
SO_2_	2834	0.0	187	15.2	11.4
NO_2_	1973	1	161	30.6	20
PM_10_	2577	12	437	137.7	57.7

#### Sulfur dioxide

**Fig. 4:**
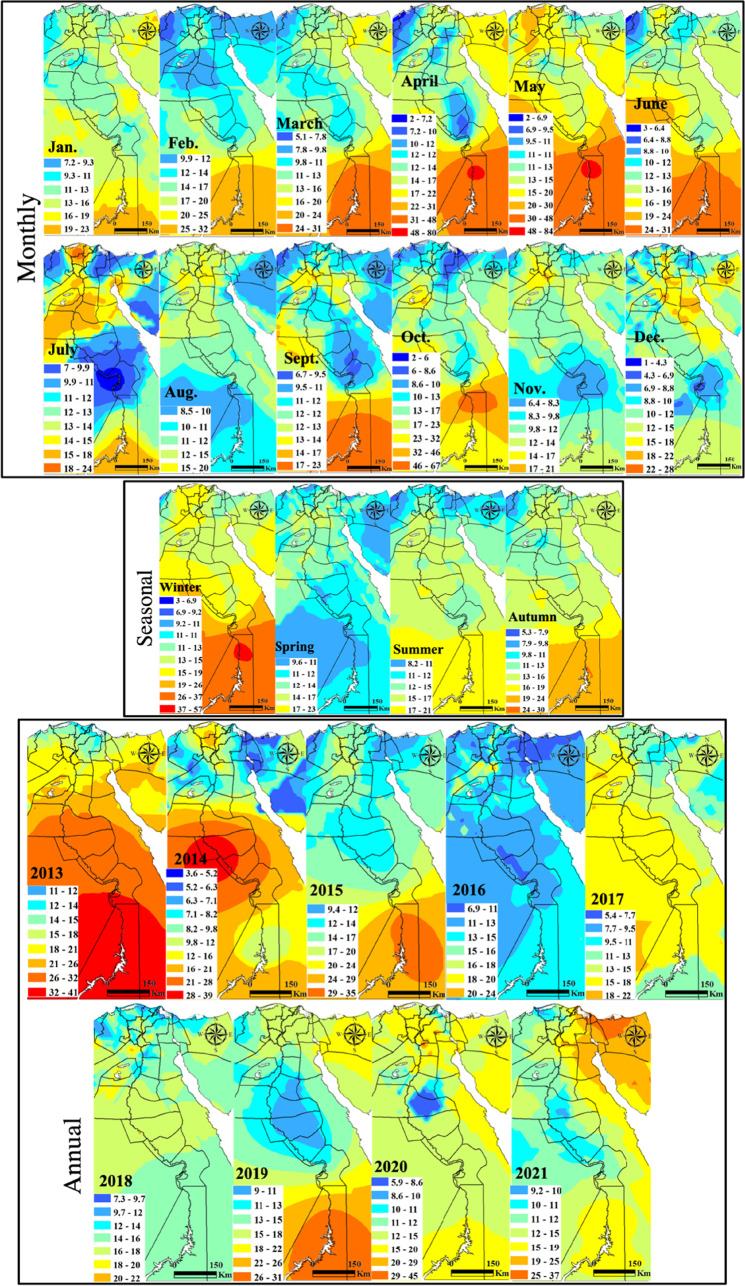
In situ SO_2_ maps of monthly, seasonal, and annual concentrations (µg m.^−3^)

Spatial distribution of SO_2_ with monthly, seasonal, and annual temporal averages over Egypt is shown on Fig. 4. The monthly mean SO_2_ concentrations varied widely during the considered time interval. An improving trend in SO_2_ concentrations was observed between the years 2013 and 2016 and fluctuated afterward, sometimes with extreme reversed trends. This improvement is mostly related to the significant increase in the natural gas demand for vehicles alternating benzene and low-sulfur fuel strict policies. SO_2_ limits, according to the Egyptian Environmental Law 4/1994 amended in Law 9/2009 and Law 105/2015, are 50 μg m^−3^ and 60 μg m^−3^ for urban and industrial areas, respectively. Local plumes with SO_2_ range levels of 48–84 μg m^−3^ mark the southern Upper Egypt stations of Idfu, Kom Ombu, and Aswan of common occurrence in April and May. These stations attained the largest average range levels of 37–57 μg m^−3^ in winter with the largest plume at a range level of 32–41 μg m^−3^ occurred in 2013. SO_2_ local plumes in the level ranges of 28–39 μg m^−3^ dominated in Assut, Sohag, and Nagaa Hammadi in 2014. SO_2_ high levels in these cities are attributed to sulfur-rich fuel burning emissions from the Nile cruises along with electricity generators for domestic and industrial uses in the newly developed industrial zones. In general, there is a regional decrease in 2020 of SO_2_. The marked increase of SO_2_ in 2021 in the Suez Canal economic zone (SCZone) stations compared to that of 2019 confirms that the IMO (2020b) new regulations started on January 2020 of the SO_2_ limit of 0.50 wt% from the previous 3.5wt% either were not effective or have not been strictly applied.

Significant increasing trends showed SO_2_ annual rate of change in the range level of 1.12–5 μg m^−3^ year^−1^ (Table S1). Suez attained the largest increasing rate at 5 μg m^−3^ year^−1^ followed by Mansoura1 of 3 μgm ^−3^ year^−1^. The large rate of increasing SO_2_ in Suez is attributed to the ever-increasing number of ships passing daily in the Suez Canal with emission sourced from ships’ sulfuric fuel consumption. Mansoura1 is an ever-evolving industrial city with many factories and dense vehicles on roads, where fuel burning is a major source of SO_2_. Significant improving trends varied in rates from –0.4 in Alexandria to –2.6 μg m^−3^ year^−1^ in Shubra El-Kheima. Improving air quality in these cities is related to climate conditions such as in Alexandria which is a coastal city with low pollution loads or for governmental regulations set for industrial areas such as Shubra El-Kheima or in crowded cities such as in Al-Kolaly and Nasr City. Homogeneity tests of SO_2_ are shown on Table S2. Homogeneity tests of SO_2_ indicated thirteen inhomogeneous time series data, which later have been differentiated into significant five decreasing and eight increasing trends. Inflection points (*t*) where significant abrupt increase or decrease changes occurred varied between May and December for years ranging from 2014 to 2019.

**Fig. 5:**
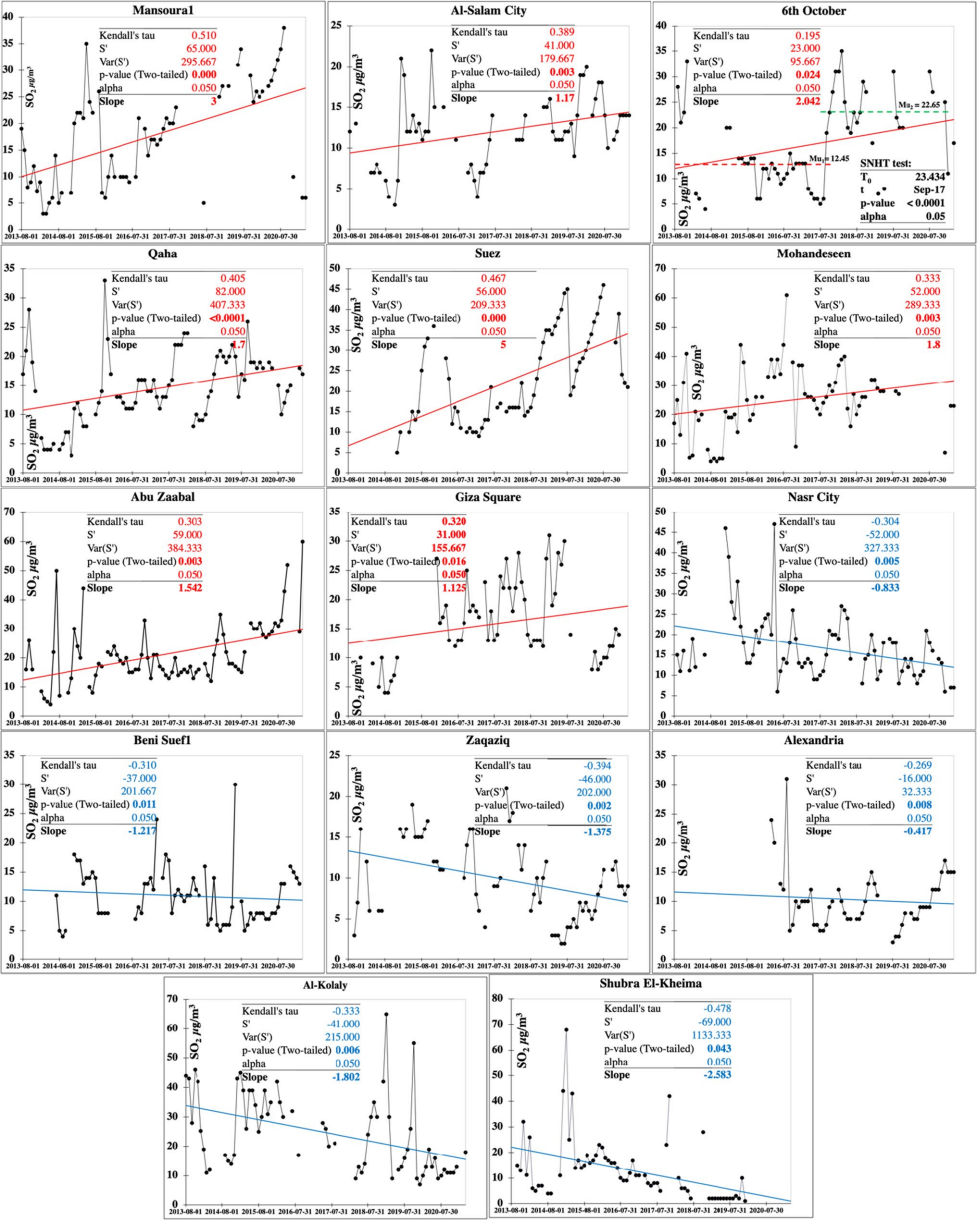
Monitoring stations attaining significant increasing (red) and decreasing (blue) Mann–Kendall trends and their slopes for the SO_2_ pollutant (µg m.^−3^)

The characteristics of the seasonal Mann–Kendall trends and their Sen’s slope referring to the SO_2_ annual rate of change are shown on Fig. 5. Eight stations attained the most significant increasing trends with the largest annual rate of change including Suez, Mansoura1, 6^th^ October, Mohandeseen, Qaha, Abu Zaabal, Al-Salam City, and Giza Square, arranged in a decreasing rate of change. Six stations showed significant improving trends arranged in decreasing order as Shubra El-Kheima, Al-Kolaly, Zaqaziq, Beni Suef1, Nasr City, and Alexandria. Suez clarified the largest recorded annual change rate at 5 µg m^−3^ while the most improved annual rate trend at –2.6 µg m^−3^ marked Shubra El-Kheima.

**Fig. 6:**
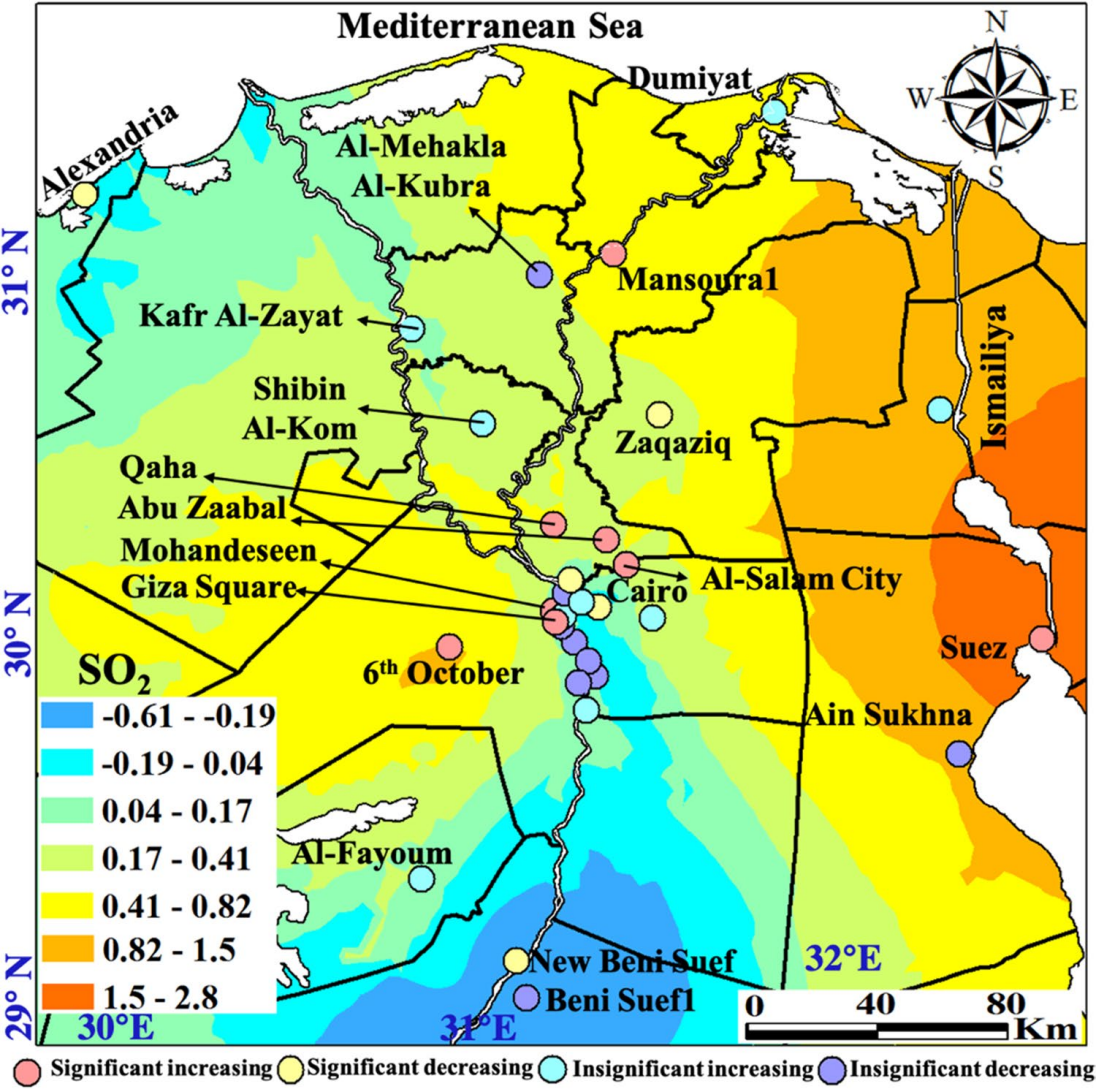
Spatial distribution of the detected monotonic trends annual change rate of the SO_2_ pollutant (µg m.^−3^)

Monotonic trends were only detected for monitoring stations located at Beni Suef and northwards (Fig. 6). SO_2_ plume areas with significantly increasing trends at the largest annual rate in the range level of 0.82–2.8 µg/m^3^ mark the SCZone cities of Suez, Ismailiya, and Port Said. These values are lower than those in Table S1 since the krigged surface of the trend pattern is an approximation and does not honor the exact values at the stations. The ship traffics of large number of varying cargo types passing the Suez Canal are the main source of SO_2_ pollution in this region.

#### Nitrogen dioxide

**Fig. 7:**
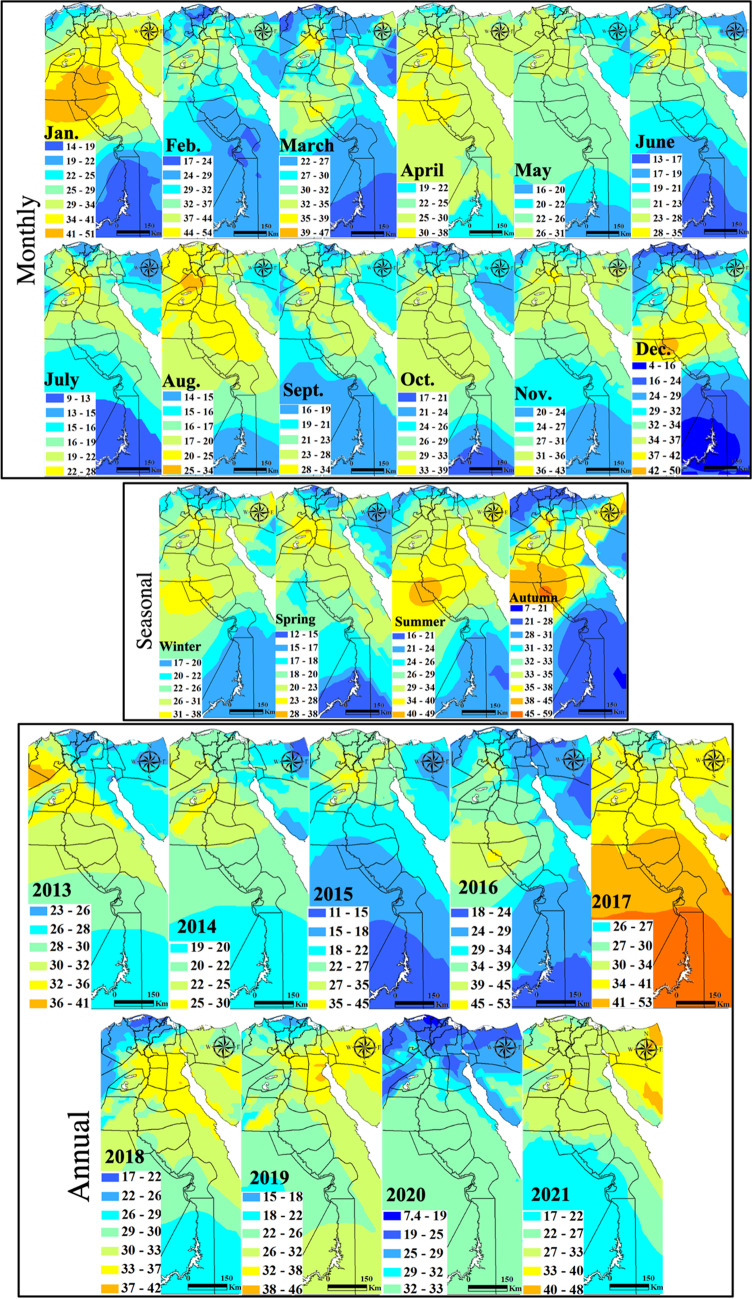
In situ NO_2_ maps of monthly, seasonal, and annual concentrations (µg m.^−3^)

**Fig. 8:**
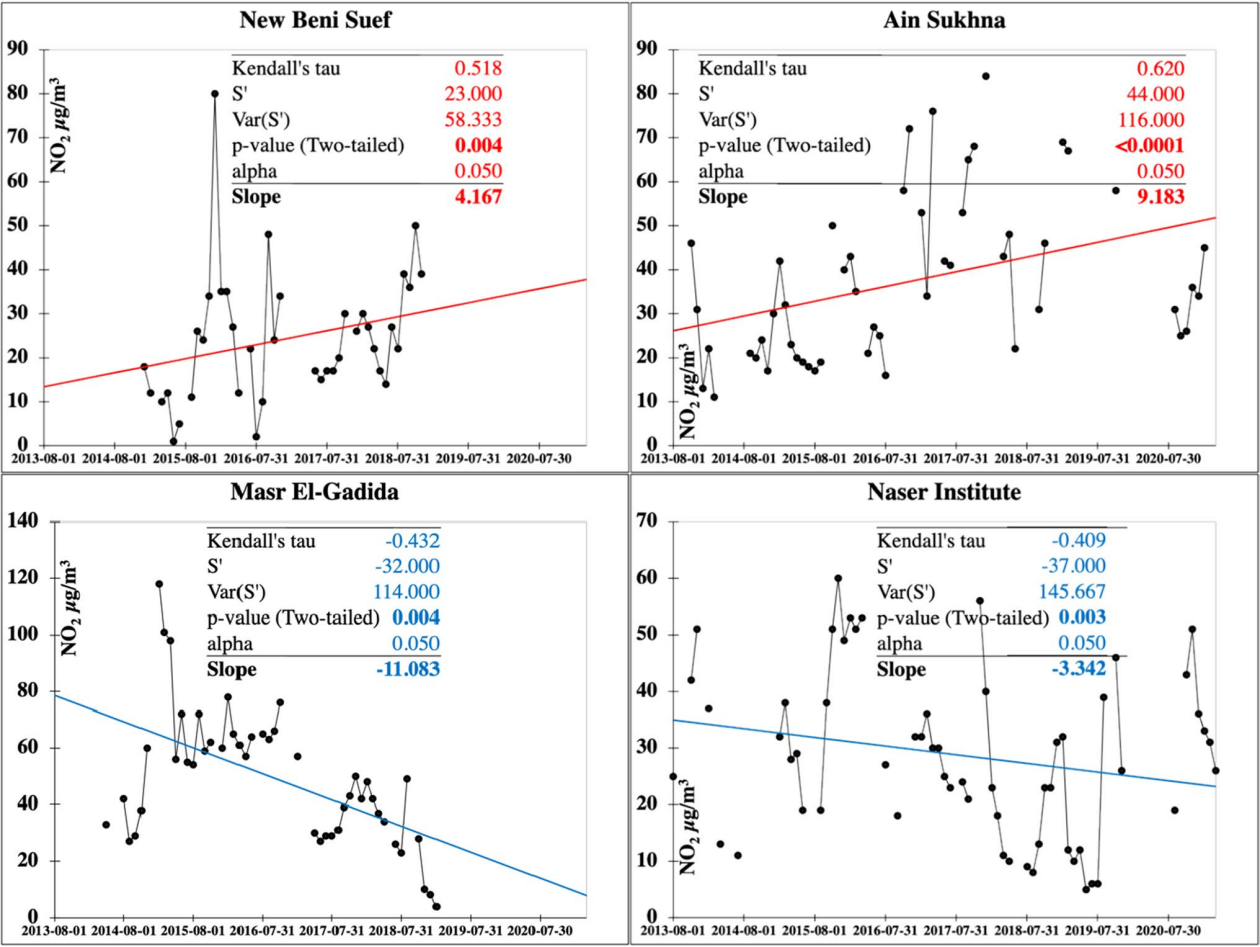
Monitoring stations attaining significant increasing (red) and decreasing (blue) Mann–Kendall trends and their slopes for the NO_2_ pollutant (µg m.^−3^)

The NO_2_ is mostly emitted via combustions including diesel and gasoline fuel engines, and industrial activities with fossil fuel combustion marked as the main source of NO_2_ in the Middle East region (Lelieveld et al. 2015). NO_2_ annual limits, according to the Egyptian standards*,* are of 60 μg m^−3^ and 80 μg m^−3^ for urban and industrial areas, respectively. Spatial distribution of NO_2_ with monthly, seasonal, and annual temporal averages over Egypt is shown on Fig. 7. Local plumes with NO_2_ range levels of 41–51 μg m^−3^ dominate in December and January between Minia and Assut, while a plume of NO_2_ pollution marks GCMA stations in August. NO_2_ pollution is much stronger in summer and autumn with plume range of 40–59 μg m^−3^ dominating in Assut stations. NO_2_ was much active in 2017 in the Upper Egypt stations from Assut and southward mutually associated with the SO_2_ pollutant. The characteristics of the seasonal Mann–Kendall trends and their Sen’s slope of NO_2_ are shown on Fig. 8 with the annual change rate shown on Table S3. Ain Sukhna located on the Gulf of Suez clarified the largest increasing rate of change of about 9.2 μg m^−3^ year^−1^ followed by New Beni Suef City with a rate of 4.17 μg m^−3^ year^−1^. Significant improving trends with the largest rates marked Cairo districts of Masr El-Gadida (–11.08 μg m^−3^ year^−1^) and Naser Institute (–3.34 μg m^−3^ year^−1^).

**Fig. 9:**
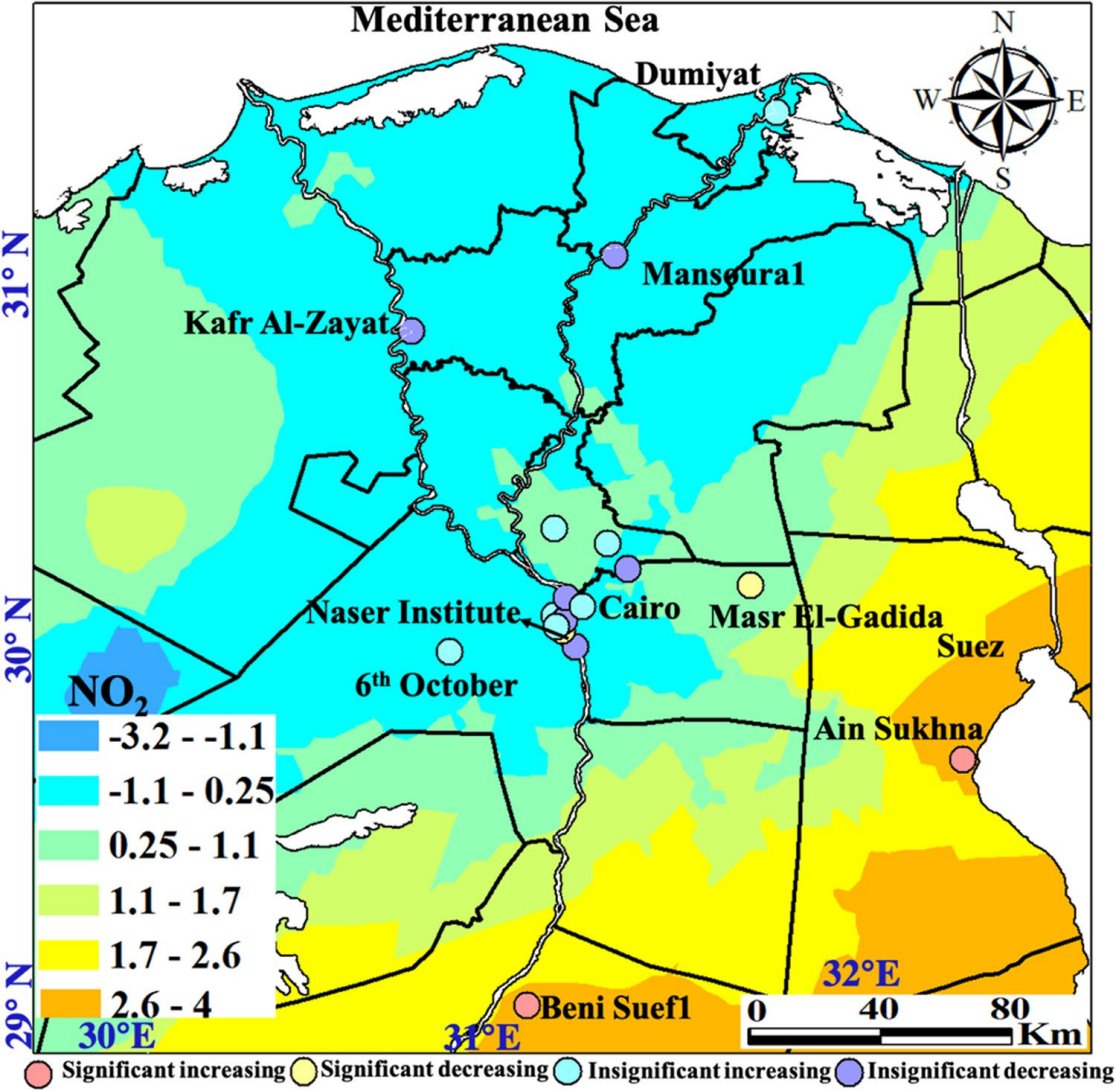
Spatial distribution of the detected monotonic trends annual change rate of the NO_2_ pollutant (µg m.^−3^)

NO_2_ plume areas in Suez and Beni Suef1 are of remarkable occurrence with significantly increasing trends at the largest annual change rate in the range level of 1.7–4 µg m^−3^ (Fig. 9).

The improving trend was sustained in 2013–2016 for NO_2_ as for SO_2_, with 2020 being the most improved NO_2_ concentration in Egypt where Greater Cairo and the Nile Delta are the most improved being the most populous regions that have been locked following COVID-19 in 2019. Seasonal variation showed improvement from winter to spring and then reversed to increase in summer to reach its maximum in autumn, mostly related to the fact that in winter and spring, NO_2_ is less affected by meteorological conditions (e.g., inversion and the stable boundary layer are dominant) and chemical mechanisms (e.g., photolysis, which is dominant in summer and autumn; Torbatian et al. 2020). Homogeneity tests of NO_2_ are shown in Table S4. Homogeneity tests indicated only four inhomogeneous time series data: significant two decreasing and two increasing trends. Inflection points (*t*) the winter months of January 2018 and February 2017, and the autumn months of August 2016 and September 2015 for the increasing trends.

#### ***Particulate matter (PM***_***10***_***)***

**Fig. 10:**
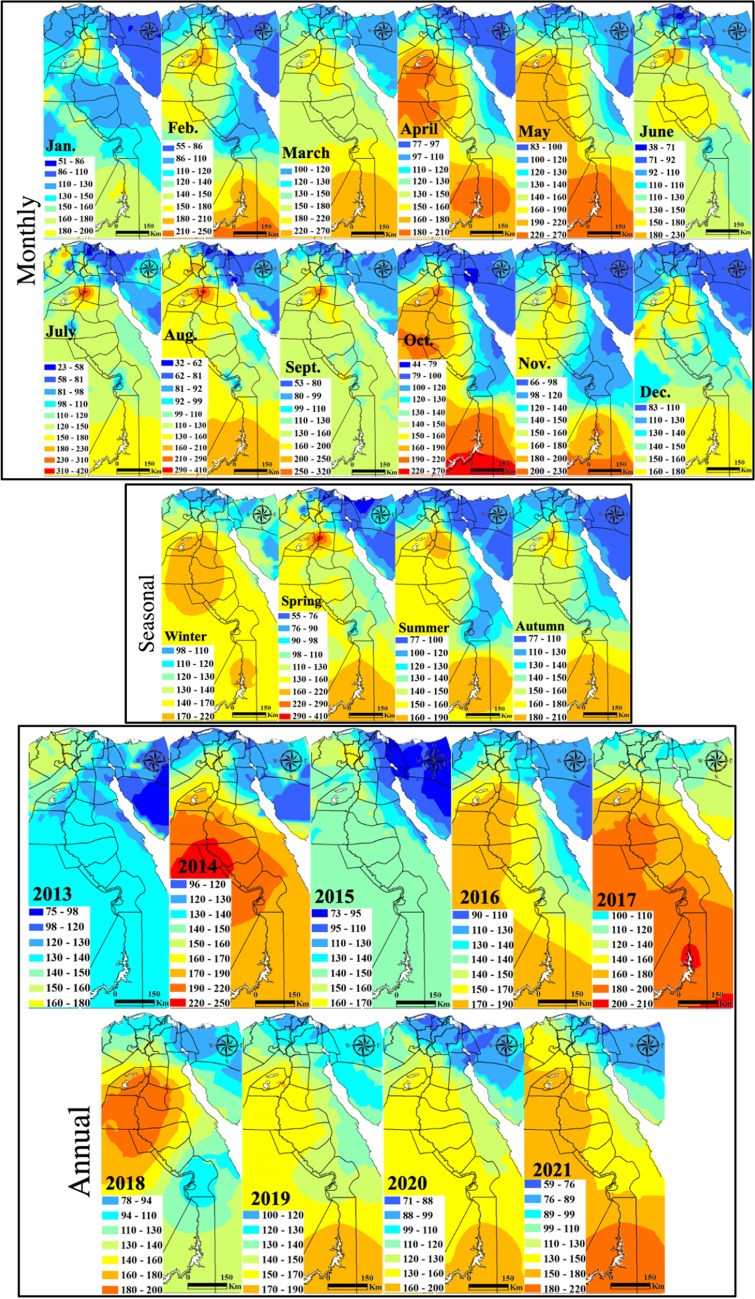
In situ PM_10_ maps of monthly, seasonal, and annual concentrations (µg m.^−3^)

**Fig. 11:**
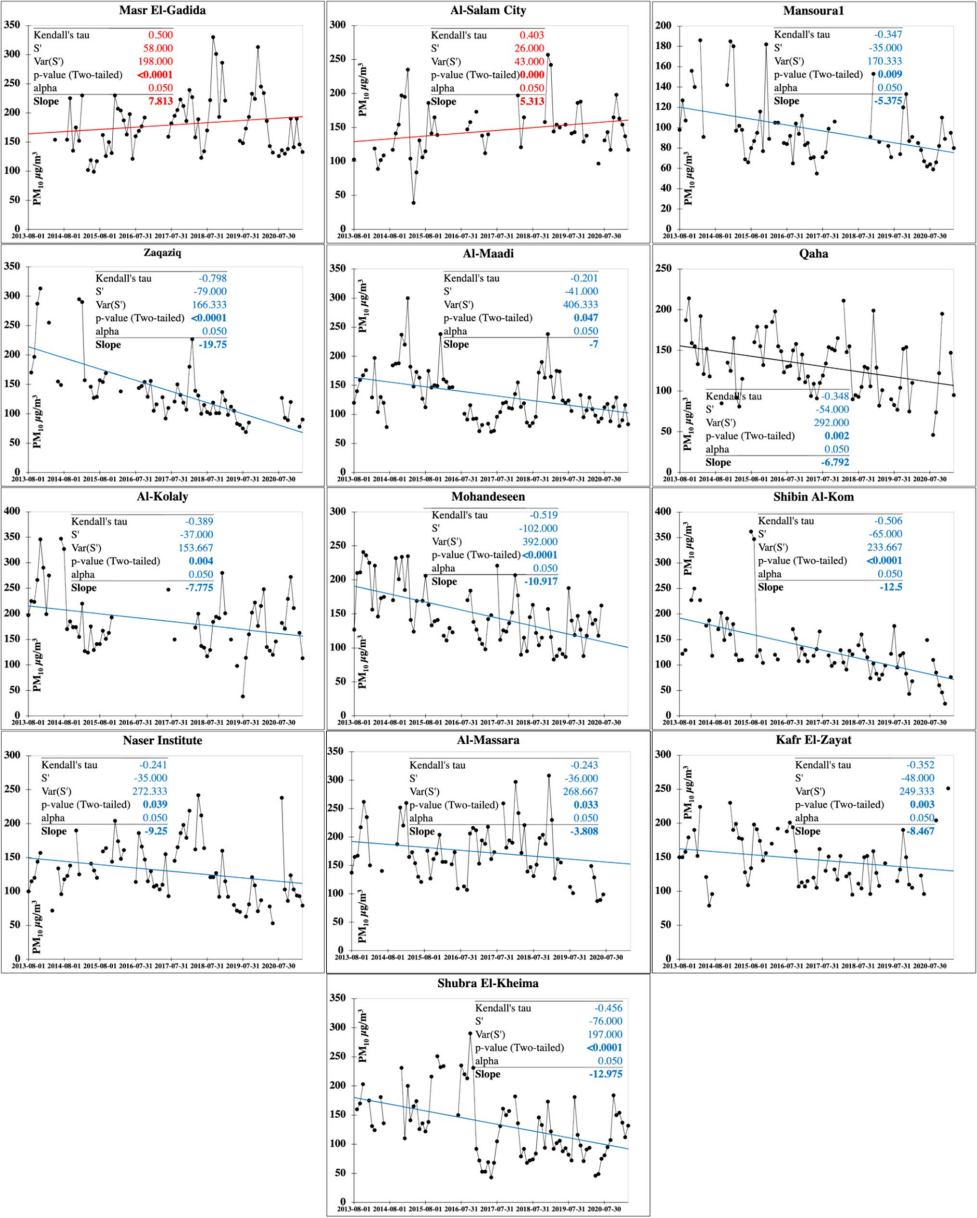
Monitoring stations attaining significant increasing (red) and decreasing (blue) Mann–Kendall trends and their slopes for the PM_10_ pollutant (µg m.^−3^)

The spatial distribution of PM_10_ with monthly, seasonal, and annual temporal averages over Egypt is shown on Fig. 10. Local standards for the PM_10_ annual limits are 70 μg m^−3^ and 80 μg m^−3^ for urban and industrial areas, respectively. Two significant plumes are widespread over GCMA and Aswan from February to November every year during the monitoring period, but reach the maximum in July with levels of 310–420 μg m^−3^. These plume areas continue in all seasons but of much strong effect in the spring (290–410 μg m^−3^) that dominated in 2014 and 2017. PM_10_ showed a regional decrease in 2020 following the COVID-19 lockdown. The characteristics of the seasonal Mann–Kendall trends and their PM annual rate of change are shown in Fig. 11 and Table S5. Many GCMA districts clarified the largest rates of increase in PM_10_ of which Masr El-Gadida and Al-Salam City attained the largest significant rate of 7.81 μg m^−3^ year^−1^ and 5.31 μg m^−3^ year^−1^, respectively. Significantly improving areas of Zaqaziq, Shubra El-Kheima, and Mohandseen clarified the largest rates of − 19.75 μg m^−3^ year^−1^, − 12.97 μg m^−3^ year^−1^, and − 10.92 μg m^−3^ year^−1^, respectively. Recent improving PM concentrations in the GCMA are attributed to the control of kiln dust emitted from cement industries through the use of electrostatic precipitators such as in the Helwan industrial district.

Homogeneity tests of PM_10_ are shown on Table S6. Homogeneity tests indicated thirteen inhomogeneous time series data: significant eleven decreasing and two increasing trends. Inflection points (*t*) marked August and December 2015 for the increasing trends mostly associated with wind-blown desert dust and heavy traffic and industry. Marked abrupt change varied between May and November common in 2016 and 2018 for the decreasing trends, mostly of climatic origin associated with the calm end of spring and Autumn.

**Fig. 12:**
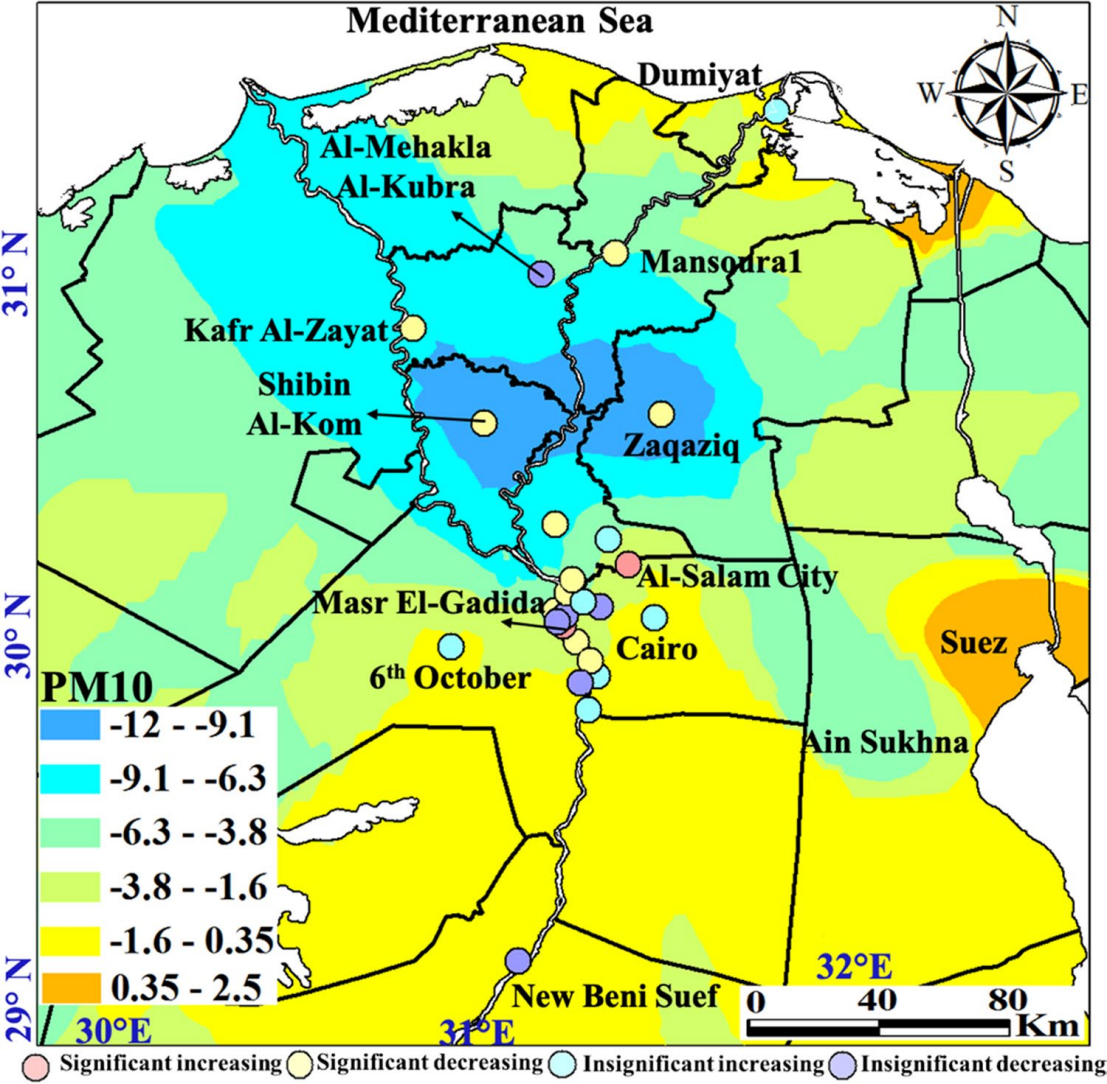
Spatial distribution of the detected monotonic trends annual change rate of the PM_10_ (µg m.^−3^)

PM_10_ plume areas with significantly increasing trends at the largest annual change rate in the range level of 0.35–2.5 µg m^−3^ mark the Suez Canal economic zone cities of Suez and Port Said (Fig. 12).

Overall, the impact of dust on the annual average PM_10_ has a clear latitudinal gradient (from 70 to greater than 220 µg m^−3^ going from north to south); this feature is mainly driven by an increased number of dust episodes per year with decreasing latitude.

The monthly and seasonal averages of dust-PM_10_ (55–220 µg m^−3^) are more homogenous over the country with peak plumes south of GCMA and over Aswan, shown to be mainly influenced by the site type, with enhanced values in more urbanized locations. A similar PM_10_ gradient but at lesser magnitudes has affected Italy by atmospheric transport of desert dust from the Northern Africa and the Middle East drylands (Barnaba et al. 2022). Also, high Saharan-dust PM_10_ background levels are recorded in Southern Spain (30 µg m^−3^ PM_10_ as an annual mean for rural areas) and very similar values are recorded in industrial and urban areas (Rodriguez et al. 2001). Several desert dust episodes affected atmospheric aerosols in the planetary boundary layer over Portugal in 2016 (Gama et al. 2020).

Our research confirmed the dominance of the PM_10_ dust loadings in the spring with plumes focused over south GCMA and Aswan. This conclusion agrees well with the results of Moulin et al. (1998) where in the EM basin (extending from Turkey to northern Africa and eastward to Iran), dust loadings are commonly due to the occurrence of intense Sharav cyclones, which are generated by the thermal contrast between cold Atlantic air and warm continental air, over the south of the Atlas Mountains (Morocco).

Large contributors to natural PM levels in Egypt are the northeasterly winds in spring and the fresh-to-strong hot “Khamasin” southerly wind which are usually loaded with high levels of natural sand and dust. Anthropogenic sources of PM emission are especially from incomplete combustion processes, heavy oil industrial activities (iron and steel, cement, brick industry, ceramics, coke plants, etc.), and traffic, about 88% of which comes from old and poorly maintained cars and buses with emissions estimated to be 1800 tonnes in 2000, a more than sevenfold increase since 1980 only in the GCMA (Nasralla 2001).

### Validation and anthropogenic impact of air pollutants

**Fig. 13:**
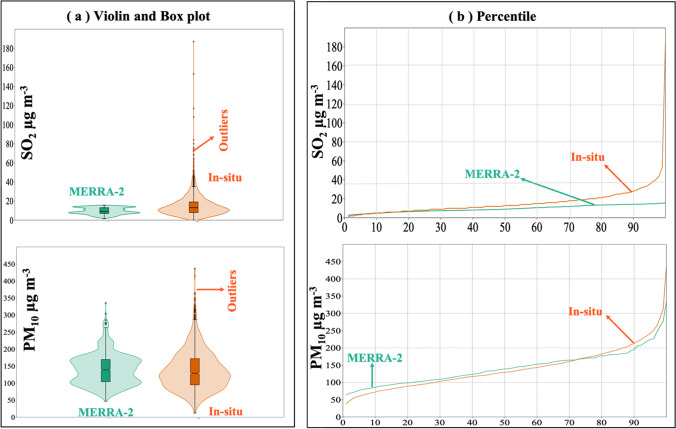
Plots of **a** Violin and Box of the MERRA-2 air pollutants, plots of in situ against MERRA-2 SO_2_ and PM_10_, and **b** percentile

Comparison of the MERRA-2 to the in situ data is carried out by investigating the descriptive statistics, Pearson’s coefficients of correlation and determination, and the root mean squared error (RMSE). For the MERRA-2 and the in situ data, respectively, descriptive statistics of the air pollutants averages at all stations showed wide range levels of 3–13 and 2–31 for SO_2_, and 80–178 and 68–329 for PM_10_, but with close averages of 10 and 15 for SO_2_, and 142 and 133 for PM_10_ (Table 6). Violin and Box along with the percentile plots (Fig. 13) clarified close averages but the in situ data showed much more outliers. SO_2_ errors are lowest for values less than 20 g/m^3^, above which they gradually increase. The PM_10_ error is lowest for values less than 300 g/m^3^ and gradually increases at larger concentrations.

**Table 6:** Descriptive statistics of averages of MERRA-2 against the in situ air pollutants at all stations

	Variable	Minimum	Maximum	Mean	Std. deviation
MERRA-2	SO_2_	3	13	10	3
	CO	81	185	149	35
	O_3_	271	296	288	5
	PM_1_	12	81	48	28
	PM_2.5_	52	99	75	12
	PM_10_	80	178	142	32
In-situ	SO_2_	2	31	15	7
	NO_2_	8	77	30	14
	PM_10_	68	329	133	48

**Fig. 14:**
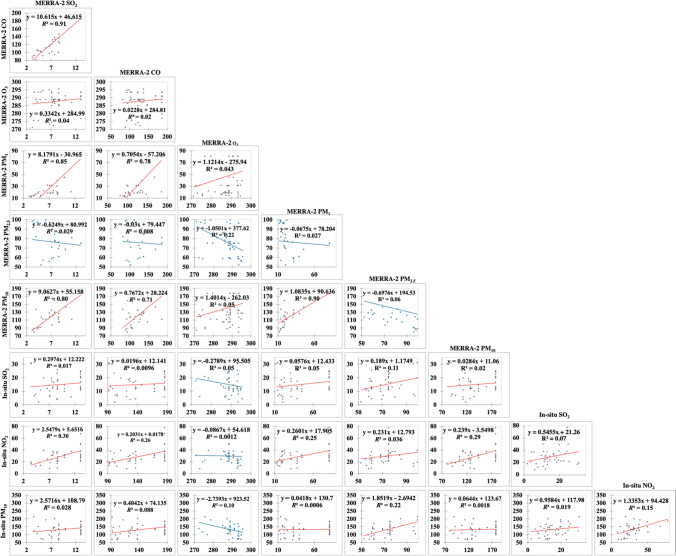
Regression analysis of air pollutants for the MERRA-2 against the in situ data

Pearson’s correlation coefficients and best-fitted determination coefficients of MERRA-2 against the in situ air pollutants averages at all stations are shown in Table 7, with empirical equations shown in Fig. 14. The SO_2_ and the PM_10_ MERRA-2 contents did not correlate well with their in situ counterparts. However, at a significance level of 99%, results of MERRA-2 confirm the co-existence of SO_2_ and CO (*r* = 0.95), with the largest contribution coming from PM1 (*r* = 0.92) and PM_10_ (*r* = 0.90) for SO_2_, and PM_1_ (*r* = 0.88) and PM_10_ (*r* = 0.84) for CO. Tropospheric ozone significantly decreases in elevated PM_2.5_ air content (*r* =  − 0.47). PM_1_ and PM_10_ coexist (*r* = 0.95). The in situ data clarified the moderate contribution of NO_2_ to the PM_10_ concentration. Also, the in situ NO_2_ showed strong positive correlation (*r* > 0.50) with most of MERRA-2 air pollutants except ozone, while fairly correlated with PM_2.5_ (*r* = 0.20). Also, at a significance level of 99%, SO_2_ air content can also be identified empirically at an accuracy of 91%, 85%, and 80% from CO, PM_1_, and PM_10_, respectively. CO fitted linearly at an accuracy of 78% and 71% from PM_1_ and PM_10_ contents, respectively. PM_1_ concentration can be estimated from PM_10_ content (*R*^2^ = 0.91). Also, the in situ NO_2_ concentration can be moderately approximated from the MERRA-2 SO_2_ and PM_10_ (*R*^2^ = 0.91).

**Table 7:** Pearson’s correlation coefficients and best-fitted determination coefficients of MERRA-2 against the in situ air pollutants averages at all stations

		Variables	MERRA-2						In situ		
			SO_2_	CO	O_3_	PM_1_	PM_2.5_	PM_10_	SO_2_	NO_2_	PM_10_
*r*	MERRA-2	SO_2_	1								
		CO	*0.95*	1							
		O_3_	0.20	0.15	1						
		PM_1_	*0.92*	*0.88*	0.21	1					
		PM_2.5_	− 0.17	− 0.09	* − 0.47*	− 0.16	1				
		PM_10_	*0.90*	*0.84*	0.23	*0.95*	− 0.25	1			
	In-situ	SO_2_	0.13	0.10	− 0.22	0.22	0.33	0.13	1		
		NO_2_	*0.55*	*0.51*	− 0.03	*0.50*	0.20	*0.54*	0.27	1	
		PM_10_	0.17	0.30	− 0.31	0.02	0.47	0.04	0.14	0.40	1
*R*^2^	MERRA-2	SO_2_	1								
		CO	*0.91*	1							
		O_3_	0.04	0.02	1						
		PM_1_	*0.85*	*0.78*	0.04	1					
		PM_2.5_	0.03	0.01	0.22	0.03	1				
		PM_10_	*0.80*	*0.71*	0.05	*0.91*	0.06	1			
	In-situ	SO_2_	0.02	0.01	0.05	0.05	0.11	0.02	1		
		NO_2_	0.30	0.26	0.00	0.25	0.04	0.30	0.07	1	
		PM_10_	0.03	0.09	0.10	0.00	0.22	0.00	0.02	0.15	1

Strong correlation coefficients greater than 0.5 are shown in italics

**Fig. 15:**
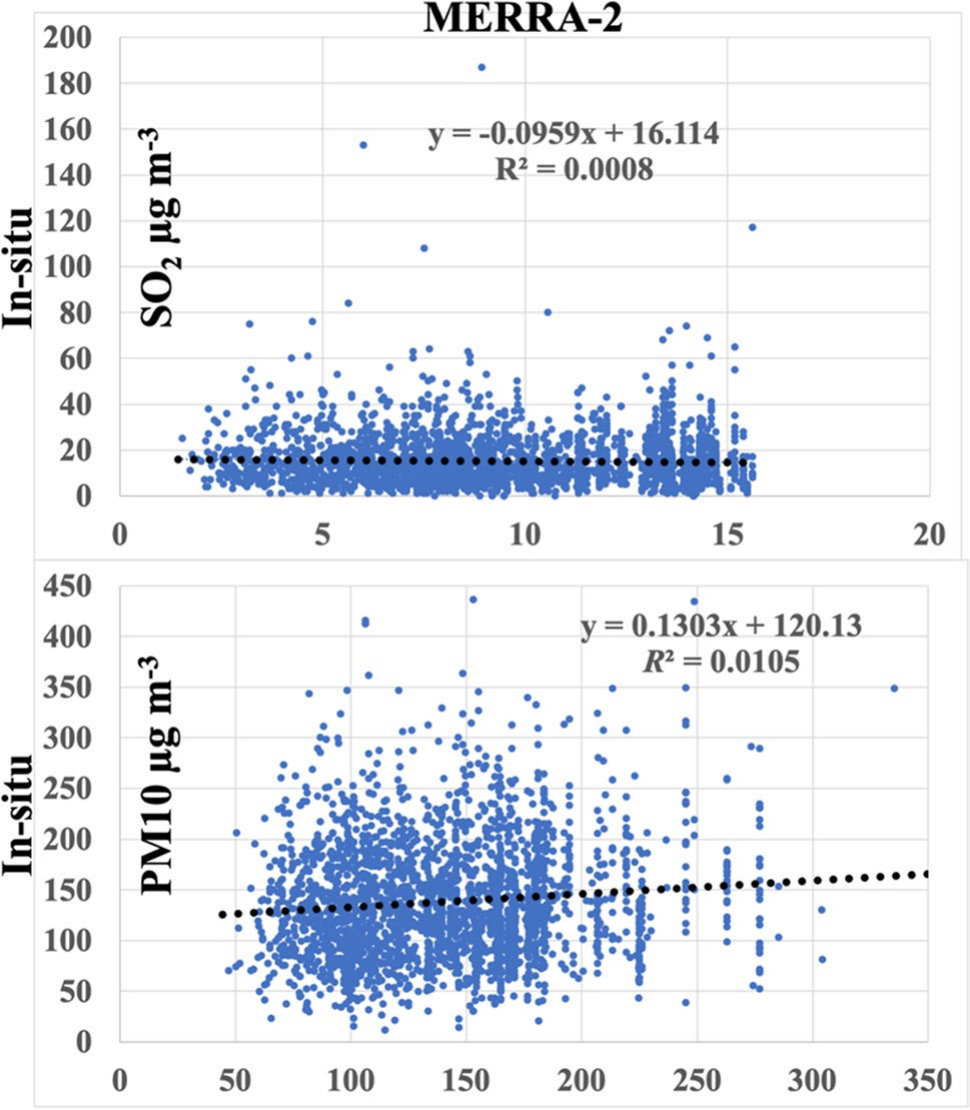
Regression analysis of MERRA-2 against the in situ for the SO_2_ and PM_10_ of all pairwise records at all stations

Regression analysis of MERRA-2 against in situ measured SO_2_ and PM_10_ air pollutants pairwisely recorded is shown in Table 8 and Fig. 15 achieved very low monthly cross-validation *R*^2^ values with RMSE of 13.38 µg m^−3^ and 69.46 µg m^−3^, respectively. This confirmed a significant underestimation of the high aerosol loading phenomenon of MERRA-2 AOD products which agrees well with the result of a long-term analysis of China’s economically developed regions compared to that with MODIS (Song et al. 2018).

**Table 8:** Evaluation of the pairwise records of the MERRA-2 against the in situ SO_2_ and PM10 air pollutants

	SO_2_ (*n* = 2824)		PM_10_ (*n* = 2578)	
	In situ	MERRA-2	In situ	MERRA-2
Min	0	1.53	12	47
Max	187	15.61	437	335
Mean	15.23	9.50	138	141
Std. dev	11	3.46	58	45
Median	13	9	129	139
25% percentile	8	7	95	104
75% percentile	19	13	172	169
Coeff. var	74	36	42	32
RMSE	13.38		69.46

**Fig. 16:**
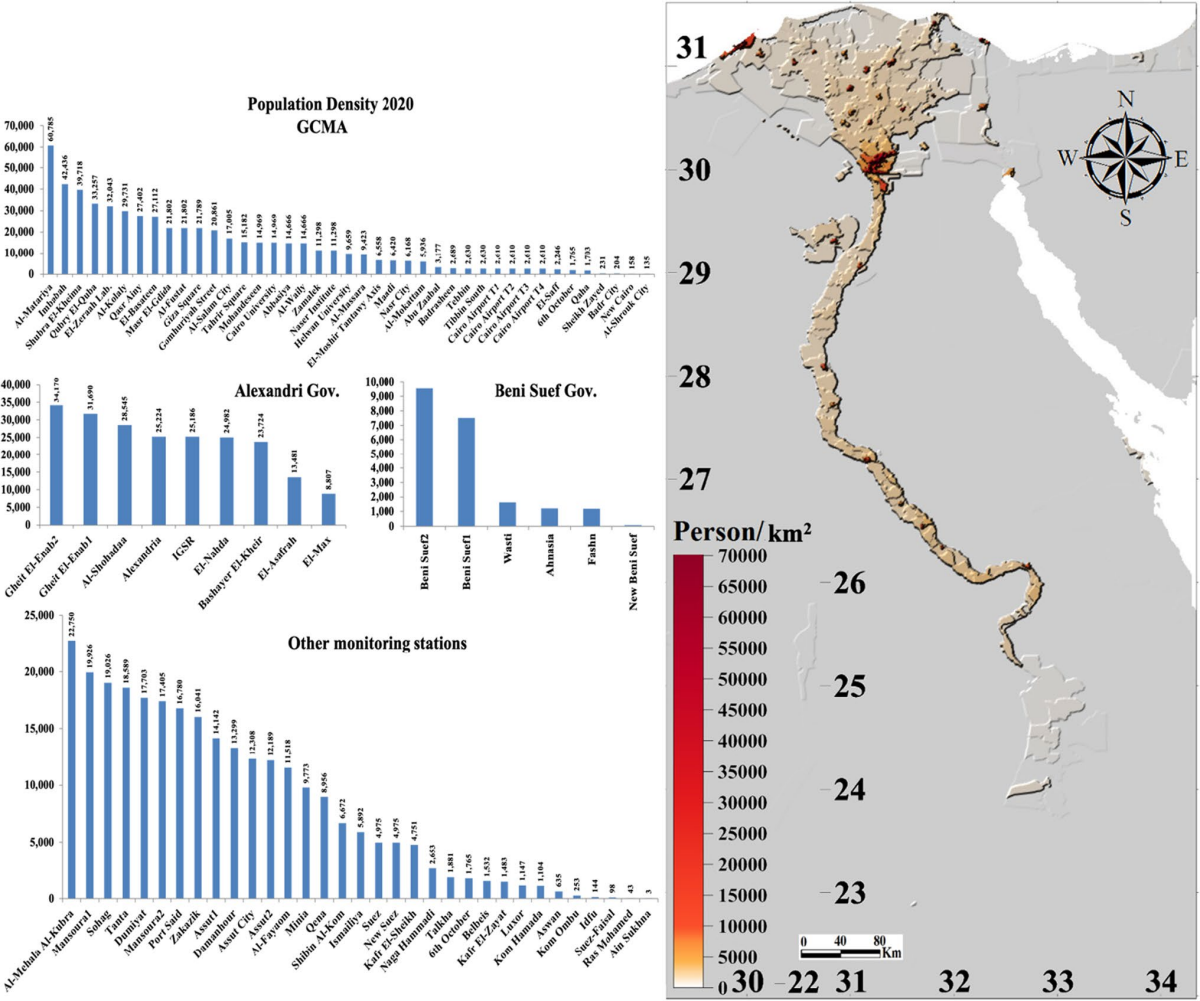
Population density as of 2020 of the GWpv4.11 at 1-km resolution; histogram (left) and heat map (right)

Population density at the monitored stations showed an average of 11,600 person/km^2^, mostly concentrated in the Nile Valley and Delta regions (Fig. 16). In general, GCMA is the most populous area, followed by Alexandria and El-Mehala Al-Kubra. The districts of Al-Matariya, Imbabah, and Shubra El-Kheima in GCMA recorded the top three most populated areas of Egypt reaching about 61,000, 42,000, and 40,000 person/km^2^, respectively, followed by Gheit El-Enab2 in Alexandria (34,000 person/km^2^). El-Mehala Al-Kubra recorded the highest mean density of 22,700 people per square kilometer in the Middle Delta region. Ras Mohamed in South Sinai and Ain Sukhna in Suez revealed the least dense areas of 43 and 3 person/km^2^, respectively.

**Fig. 17:**
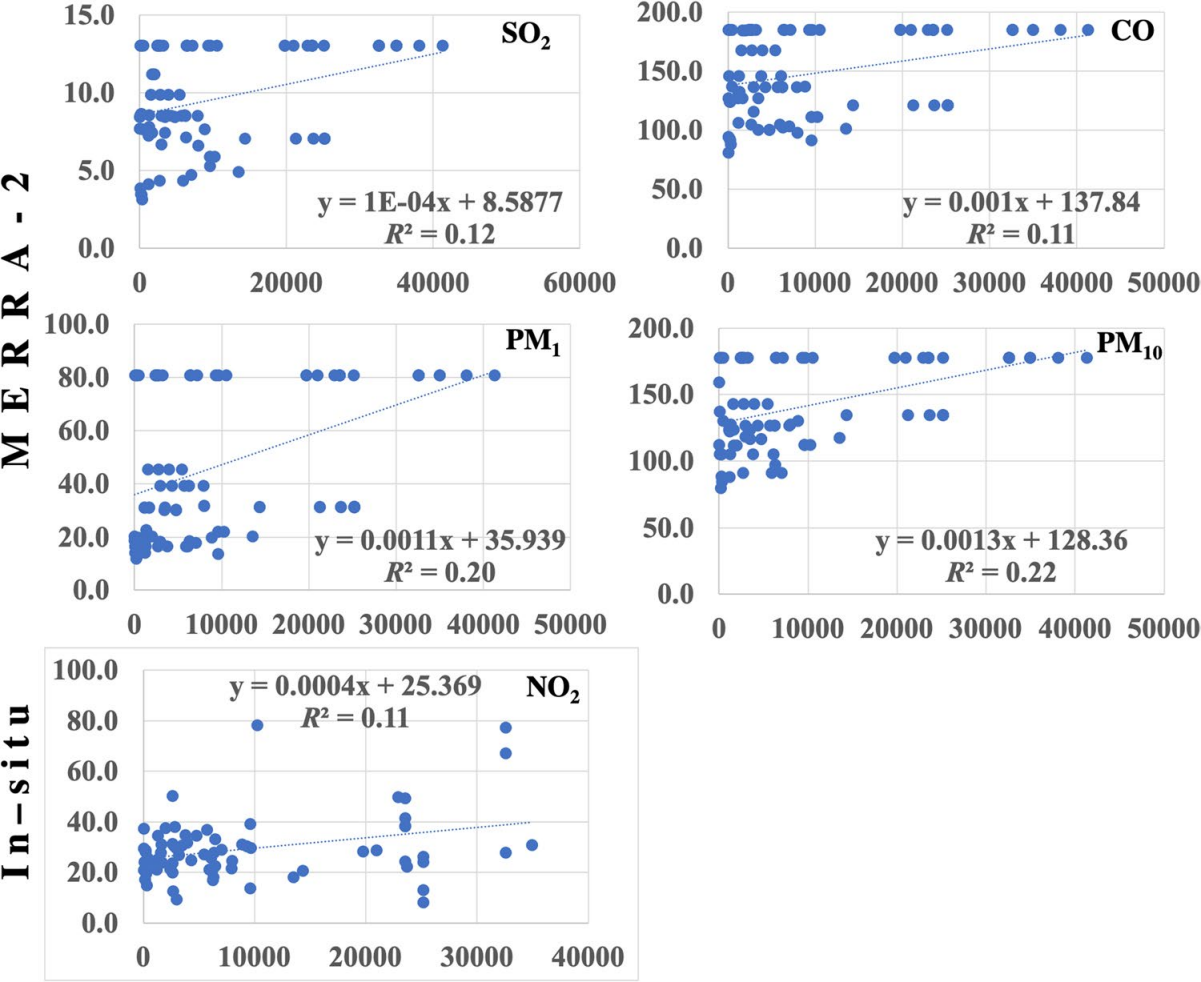
Pearson’s coefficients of best-fit linear determination (*R*^2^) of the MERRA-2 and the in situ air pollutant averages against population density at all stations in the monitored period

Population density showed larger correlation and determination coefficients at 5 km than at other resolutions (Table 9). The population density at 5 km correlated well, at a significance level of 99%, with the MERRA-2 PM_10_ (*r* = 0.46), PM_1_ (*r* = 0.45), SO_2_ (*r* = 0.35), and CO (*r* = 0.35), in decreasing order, and also the in situ NO_2_ (*r* = 32). Empirical equations for the regression analysis are shown in Fig. 17. Best-fit linear regression revealed anthropogenic inputs of 22%, 20%, 12%, and 11% for the concentrations of MERRA-2 PM_10_, PM_1_, SO_2_, and CO, respectively, and 11% for in situ NO_2_.

**Table 9:**
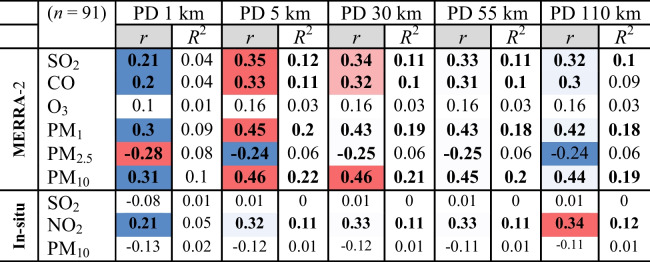
Pearson’s coefficient of correlation (*r*) and determination (*R*^2^) of MERRA-2 and the in situ air pollutants averages against population density at all stations

At a significance level of 99%, values are highlighted from lowest (blue) to largest (red) where PD is the population density/km.^2^ as of 1 July 2020

MERRA-2 PM_2.5_ exhibited the largest negative correlation coefficient (*r* =  − 0.28) at 1-km resolution indicating local anthropogenic impact while the in situ NO_2_ showed a gradual increase at coarser scales, being maximum at 110 km (*r* = 0.34) indicating a regional anthropogenic impact.

## Discussion

### Sustaining air pollution trends over the industrial zones

#### GCMA industrial zone

As shown in the in situ data trends, the 6th October city recorded the highest indoor SO_2_ levels of 2.042 μg m^−3^ year^−1^ followed by Mohandeseen at 1.8 μg m^−3^ year^−1^ and Abu Zaabal at 1.5 μg m^−3^ year^−1^, and Suez at 5 μg m^−3^ year^−1^ and Mansoura1 at 3 μg m^−3^ year^−1^. There was a NO_2_ trend of 3.25 μg m^−3^ year^−1^ in Mohandeseen, 2.375 at Giza Square, and 1.67 at Abu Zaabal, following the largest recorded over Egypt at Ain Sukhna (9.18 μg m^−3^ year^−1^) and New Beni Suef (4.17 μg m^−3^ year^−1^). Misr El-Gadida and Al-Salam City have among the highest PM10 annual content rates in GCMA with 7.81 g m^−3^ year^−1^ and 5.31 g m^−3^ year^−1^, respectively.

GCMA activities consume over 40% of Egypt’s energy yet only 18% of the country’s population (Nasralla 2001). This energy is mostly consumed by intensive air polluting industries such as cement and construction industries, to fulfill the needs for housing and public utilities as a result of population overgrowths, accompanied by an increased volume of solid waste, disposed of and burned in the open air that accounts for 36% of the total annual load of pollution in GCMA air (EEAA Reports 2006). In these reports, the largest contribution of air pollution is from industrial emissions (32%), vehicles exhausts (26%), and agricultural (especially rice straw) and open burning of waste (6%).

GCMA houses about 50% of the industrial activities, along with the generation of electricity from thermal power stations and motor vehicles all over Egypt, where haze and smoke plumes become a common phenomenon and hence has been considered the most air polluted place in the country (Nasralla 2001). In the Greater Cairo Metropolitan Area, the most developed industrial areas are located in Shoubra El Kheima in the north and in Helwan in the south. In addition, they are adjacent to residential areas in some urban districts. According to Nasralla (2001), heavy oil and natural gas are the major fuels used for power generation in GCMA, accounting for 60% and 40% respectively.

Other sources of GCMA air pollution include emissions from buses and vehicles, which account for 1.5 million vehicles. The road density in Egypt is very low (0.045 km/km^2^) compared to the worldwide average of 0.2 km/km^2^. Passenger car units (PCU) of 1,210,000 traveled GCMA during peak hours in 2010 with a high dependency on private cars (only 23% of daily trips were by public transport). The annual economic congestion cost was estimated at 47 billion LE and is expected to reach 105 billion LE by 2030. According to the Cairo Traffic Congestion Study 2014, very high congestion rates are caused by travel time delay (36%), unreliability (25%), recurrent and non-recurrent congestion (37%), and excess fuel cost and CO_2_ emissions (2%) cause a loss of 3.6% of Egypt’s total GDP and 15% of GCMA GDP per capita (Basyoni 2016).

Solid municipal waste, which amounts to 700 thousand tons to one million tons annually, and agricultural (especially rice straw) waste combustion in the open air and the fuel used in small industries and workshops are additional sources of air pollution. Burning 2000 tons per day of municipal solid waste may add 7000 tons of SO_2_ per annum to Cairo air (Nasralla 1999), also agricultural waste burning in the Nile delta valley during autumn. Moreover, the air quality of low-lying Cairo is largely affected by natural dust carried out by wind from the surrounding desert and hills.

#### Suez Canal Economic Zone (SCZone)

Heavy shipping traffic attributed to the recent strong growth in cargo throughput the Suez Canal proved the main pollution source for most highly populated areas located in the economic SCZone, including Ain Sukhna, Suez, Ismailiya, and Port Said cities. Trends from the in situ data confirmed plume areas sustaining deteriorating air quality conditions at the largest significant (99%) annual rates of 5 μg m^−3^ year^−1^ of SO_2_ at Suez and 9.2 μg m^−3^ year^−1^ NO_2_ at Ain Sukhna, as well as range level of 0.35–2.5 µg m^−3^ of PM_10_ at Suez and Port Said. The largest rates were also confirmed by the MERRA-2 data but at lower magnitudes attributed to the underestimation inherent in the MERRA-2 air quality products. In SCZone ports, ships, machinery, and port activities evolved to cope with to the ever-increasing number of vessels of different cargo types that generate a large number of greenhouse gases and fine particulate matter (Kong and Liu 2021). A recent study has shown that on a global scale, shipping emissions take up 20% of NO_X_ and 12% of SO_2_ emissions from anthropogenic sources in 2017 (McDuffie et al. 2020). The shipping industry contributes around 13% and 15% of the global anthropogenic emissions of SO_2_ and NO_2_, respectively, which contributes to acid rain and eutrophication damaging the coastal waters (Sui et al. 2020). According to Egypt’s economy profile (https://www.theglobaleconomy.com/Egypt/Port_traffic/, last accessed 15 May 2022), the number of 20-foot containers passing through the Suez Canal ports in 2000–2019 averaged 5.27 million containers with a minimum of 1.34 million containers in 2002 and a maximum of 7.9 million containers in 2014. The latest value from 2019 is 6.31 million containers. For comparison, the world average in 2019 based on 152 countries is 5.88 million containers. When petroleum fuels are combusted in ship’s engines, they emit the greenhouse gases (GHG) as oxides of sulfur, nitrogen, and carbon (Seddiek and Elgohary 2014; IMO 2020a) that move by air mass pathways of the port wind sector (Song et al. 2022), posing severe negative effects on health and the environment in the port area. These pollutants have caused over 64,000 premature deaths concentrated mostly in coastal port areas (Winnes and Fridell 2009) with ships contributing greater than 10% to the acidification of coastal areas. The Marine Environment Protection Committee (MEPC) estimated that the number of premature deaths due to SOx emissions between 2020 and 2025 would be over 570,000 (IMO 2020a). According to the EMEP status report (2019), emissions from ships account for more than 50% of NO_2_ in central parts of the Baltic Sea area as well as a substantial percentage in coastal zones, including the Baltic states (Estonia, Latvia, and Lithuania). Carbon monoxide and carbon dioxide are the main products of fuel combustion. The marine industry is responsible for 3.1% of the global annual anthropogenic CO_2_ emissions and 2.8% of the global annual anthropogenic GHG emissions (Sui et al. 2020). It was also estimated that the emissions will increase from 50 to 250% by 2050 as marine trade grows (IMO 2020b).

To reduce ship emissions, the IMO (2020a, b) (i.e., International Maritime Organization 2020 policy) regulations came into effect on 1 January 2020, limiting the sulfur content in marine bunker fuels to 0.5 wt% from the previous 3.5 wt%. The IMO (2020a, b) regulations made compulsory following an amendment to Annex VI of the International Convention for the Prevention of Pollution from Ships (MARPOL) require plenty of liquid fuels, including high energy density, specific viscosities, flash points, pour points, lubricity, stability, and availability (Vedachalam et al. 2022). These new regulations have significantly reduced SO_2_ and NH_4_^+^ concentrations around world ports (Song et al. 2022; Zhang et al. 2022a; Zhang et al. 2022b).

#### ***Aswan industrial zone***

As per the in situ data over Aswan, SO_2_ annual rates reached 0.023 μg m^−3^ year^−1^, while CO saw annual rates of 0.26 μg m^−3^ year^−1^. Also, PM_x_ exceeded 200 μg m^−3^ at all time intervals, making Aswan the most particulate matter polluted area in Egypt, in violation of the WHO annual mean limit of 15 μg m^−3^ and Egypt’s background of 70 μg m^−3^ as stipulated in the environmental law no. 4/1994 and the executive regulations were approved on October 2005.

Trends from MERRA-2 data indicated the strongest trends of PM_x_ with range levels of 0.3–0.6 μg m^−3^ year^−1^ (PM_1_), 3.9–4.6 μg m^−3^ year^−1^ (PM_2.5_), and 3.4–4.2 μg m^−3^ year^−1^ (PM_10_). Air pollution in Aswan resulted from many combined factors: recent rapid industrial development, an increase in the number of Nile cruises, desert dust, in addition to the fuel consumption for electricity from power plants and vehicle exhaust. A few examples of the air pollution sources in the Aswan Governorate include factories owned by Egyptian Chemical Industries (KIMA), Sugar and Particleboard Wood Factory in Kom Ombo (capacity of 17,000 tons/year), Edfu Sugar and Integrated Industries Company (150,000 tons/year), Idfu Ferrosilicon Factory (40,000 tons/year), El-Nasr for Mining Co., and Misr-Edfu for Pulp and Paper, and the Sugar factory in Kom Ombo (IDA 2022). In the industrial zone built southeast of Aswan at the Aswan- al-Alaqi road, on an area of about 220 feddans and including 461 production units, 566 small-scale industries are registered for maintenance of vehicles, machinery for irrigation, metal work and car services, carpentry, electrical services, textile production, food production, and tile manufacturing. Mining and quarrying in Aswan are another major source of air pollution. Egyptian Chemical Industries (KIMA) is located 5 km east of the city of Aswan. Its main products are ammonium-nitrate fertilizer (260,000 tonnes/year) and ferrosilicon used for steel production (6600 tonnes/year). NOx and ferrosilicon dust emitted from its facilities are the major air quality environmental concerns (EEAA REPORTS 2000).

Rehabilitation of Kima Fertilizers Company (Ammonia-Urea) in Aswan has been started in 2019, through the establishment of a 60-acre full industrial city in Aswan, after the aging of the current plant to produce 1220 tons of ammonia, of which 900 tons of urea are produced daily at the newly constructed Kima plant with a total of 570 thousand tons/year and 300 tons of ammonia go to the old factory to produce 120 thousand tons/year of low and high-density ammonium nitrates, and 100 thousand tons of nitrogen ammonium nitrate fertilizers (KIMA-Aswan 2019). There were 300 Nile Cruisers working between Aswan and Luxor, along the Nile River from Aswan down to Cairo (EEAA Reports 2008).

Emissions from these sources along with the desert and road dust have been reported to lead to adverse health effects (Gupta et al. 2022a, b a & b). News reports in 2019 stated that eighty-four students suffered nonlethal suffocation at a Secondary School in the Mahmoudiya waterway area due to fumes from the Old Kima factory in Aswan (Egypt Independent News report 2019). According to news reports (ARIJ 2020), the lung hospital in Kom Ombo registered 1113 patients for treatment in 2019: 180 cases of chronic obstructive respiratory disease, 74 cases of pulmonary fibrosis, and 721 patients with bronchitis, impacted by pollution from the Kom Umbo Sugar Factory. Also, in the same year, Edfu Hospital, 50 km away from the city of Kom Ombo, recorded 917 cases of lung diseases: out of which 30 cases of lung obstruction, 13 cases of pulmonary fibrosis, and 311 patients with bronchitis impacted mostly by fumes and air pollution from sugar, ferrosilicon, phosphate, and paper pulp factories in Idfu.

## Conclusions

The study offers the initial understandings from a thorough quantitative strategy to address the patterns and trends of long-term air pollution in Egypt. The study described the variations in air pollution during a 93-month period (August 2013-April 2021). With the primary goal of identifying the loads due to climatic/anthropogenic factors combined to exacerbate the health and environmental effects of air pollution, the total accumulated seasonal and annual variabilities, as well as the identification of monotonic climatic trends over Egypt, have been examined.

When MERRA-2 air quality products, with spatiotemporal coverage of the nation, overcome the drawbacks of the few and the spatiotemporal discontinuity of the in situ pollutants monitored, integration of the in situ and satellite-based reanalysis of MERRA-2 proved promising. The seasonal and annual averages showed good correlation and provided best-fit estimates at high significance and confidence levels, proving validity for decisions related to long-term change risk reduction and management (Vera et al. 2010). At local scales, i.e., at hourly, daily, and monthly intervals, natural variations can obscure the impacts of human-induced climate change (Martel et al. 2018). Also, the in situ data, being ground-truthed, clarified trends and magnitudes not inherent in the MERRA-2 counterparts. PM_10_ trends in the Upper Egypt stations were only detected by the MERRA-2 data analysis. Furthermore, the integration of results from ground-based and satellite-based tools allows for a more comprehensive understanding and adaptation of the different impacts associated with variability and change to make best-informed decisions in forecasting events and assessing conditions in near real-time to make timely decisions.

Homogeneity tests established the validity of the data for pollutant spatiotemporal loadings variability. For this, trend turning point and the potential month and year in which significant pollution increases or decreases began are determined. This is critical for the interpretation of climate and anthropogenic inputs. The presence and magnitude of trends over time enabled a better understanding of the pollution plumes distributed in space and time, and their annual rates of increase are understood. Furthermore, Egypt’s air pollution control policies have contributed to a significant reduction in AOD in most areas of the country, a trend that is particularly evident in many monitoring stations across the country that showed varying improvement rates and time stamps due to the implementation of the toughest-ever clean air measures in line with environmental legislation, where air quality is one of the key aspects of Law 4/1994, although SO_2_, CO, NO_2_, O_3_, and particulate matter (PM_x_) are still the primary pollutants with notable spatio-temporal patterns and trends in Egypt. Therefore, this study strongly advises retaining or extending stricter policies in the future for sustaining good air quality. The COVID-19 lockdown on various in situ air pollution parameters is very clear as there is a significant decrease in the annual average of all pollutants in 2020 relative to the preceding years.

With the ever-increasing housing, industrial, and shipping development along with the vehicular traffic and airborne particles affected by the varying climatic conditions of desertic regions and the generally low levels of control over emissions, the air pollution situation is sure to deteriorate further in Egypt’s major cities, and especially in the GCMA, SCZone, and Aswan. This is underpinned by the intense motor vehicle traffic and the high density of industrial activities as well as the open burning of solid waste in and around major cities such as the GCMA. Additionally, the ever-changing shipping industry, along with all the varied types of cargo moved through the SCZone, contributes to such high levels of air pollution in such an economically prosperous area. Furthermore, the increasing number of recreational boats on the Nile River northward of Aswan as well as evolving industrial activities contribute substantially to air pollution.

These findings suggest that a well-informed control strategy, based on current health and environmental expenditure goals as well as social assistance programs, can design an educated decision for the management of the country’s air quality in diverse sectors. Also, as climate change adaption strategies are examined, anthropogenic causes affecting air quality are made clear. It is important to create a control strategy that takes the degradation of air quality’s economic, social, ecological, and health repercussions into account since the trend goes higher and increases will be increasingly noticeable in the short term. With the use of these forecasts, a number of adaptable, remedial, and preventative actions may be successfully put in place, improving planning, management, and preserving better air quality across the nation.

## Supplementary Information

The supplementary materials available online include Table S1: Mann–Kendall monotonic trends and Sen’s slope (alpha = 0.05) parameters of the in-situ SO_2_ air pollutant, Table S2: Homogeneity test (two-tailed) parameters for the significant monotonic trends in the in-situ SO_2_ air pollutant, Table S3: Mann–Kendall monotonic trends and Sen’s slope (alpha = 0.05) parameters of the in-situ NO_2_ air pollutant, Table S4: Table S4: Homogeneity test (two-tailed) parameters for the significant monotonic trends in the in-situ NO_2_ air pollutant, Table S5: Mann–Kendall monotonic trends and Sen’s slope (alpha = 0.05) parameters of the in-situ PM10 air pollutant, and Table S6: Homogeneity test (two-tailed) parameters for the significant monotonic trends in the in-situ PM10 air pollutant.

Supplementary file1 (DOCX 45 KB)

## Data Availability

Data cannot be made publicly available; readers should contact the corresponding author for details.
