# Supplementary material for: Spatio-temporal patterns and trends of the air pollution integrating MERRA-2 and in situ air quality data over Egypt (2013–2021)
Source: Air Qual Atmos Health. 2023 May 19:1–28. Online ahead of print. doi: 10.1007/s11869-023-01357-6 (PMC10195670; doi:10.1007/s11869-023-01357-6)
Supplement: Supplementary file 1 — Supplementary file1 (DOCX 45 KB) [file 11869_2023_1357_MOESM1_ESM.docx]

Table S1: Mann-Kendall monotonic trends and Sen’s slope (alpha=0.05) parameters of the in-situ SO_2_ air pollutant.

| **Station** | Kendall's tau | S' | Var(S') | p-value | **Slope** |
| --- | --- | --- | --- | --- | --- |
| **Mansoura1** | 0.51 | 65 | 227.667 | **<0.0001** | 3 |
| **Ismailiya** | 0.275 | 7 | 29.667 | 0.271 | 1.333 |
| **Nasr City** | -0.304 | -52 | 327.333 | **0.005** | -0.833 |
| **Beni Suef1** | -0.31 | -37 | 201.667 | **0.011** | -1.217 |
| **New Beni Suef** | -0.295 | -20 | 99.333 | 0.057 | -0.833 |
| **Zaqaziq** | -0.394 | -46 | 202 | **0.002** | -1.375 |
| **New Cairo** | 0.122 | 17 | 163 | 0.21 | 0.6 |
| **Al-Maadi** | -0.16 | -23 | 259.667 | 0.172 | -0.642 |
| **Masr El-Gadida** | -0.121 | -11 | 143 | 0.403 | -0.188 |
| **Dumiyat** | 0.108 | 20 | 360 | 0.317 | 0.283 |
| **Al-Fayuom** | 0.039 | 2 | 72 | 0.906 | 0.5 |
| **Al-Salam City** | 0.39 | 41 | 179.67 | **0.003** | 1.167 |
| **Alexandria** | -0.27 | -16 | 32.33 | **0.008** | -0.42 |
| **6th October** | 0.195 | 23 | 95.67 | **0.024** | 2.042 |
| **Qaha** | 0.40 | 82 | 407.33 | **<0.0001** | 1.7 |
| **Al-Kolaly** | -0.33 | -41 | 215 | **0.006** | -1.80 |
| **Suez** | 0.47 | 56 | 209.33 | **0** | 5 |
| **Mohandeseen** | 0.33 | 52 | 289.33 | **0.003** | 1.8 |
| **Qasr Ainy** | 0.038 | 7 | 357 | 0.75 | 1.35 |
| **Ain Sukhna** | -0.20 | -32 | 302.67 | 0.075 | -0.5 |
| **Tebbin** | 0.102 | 10 | 161.33 | 0.48 | 0.75 |
| **Al-Mehala Al-Kubra** | -0.045 | -4 | 138 | 0.79 | -1.04 |
| **Abu Zaabal** | 0.30 | 59 | 384.33 | **0.003** | 1.54 |
| **Shibin Al-Kom** | 0.13 | 14 | 180 | 0.33 | 0.25 |
| **Helwan University** | -0.18 | -22 | 214 | 0.151 | -0.79 |
| **Naser Institute** | -0.17 | -33 | 375.66 | 0.099 | -1 |
| **Abbasiya** | 0.096 | 19 | 395 | 0.365 | 0.33 |
| **Al-Massara** | -0.184 | -36 | 390 | 0.076 | -0.46 |
| **Giza Square** | 0.32 | 31 | 155.66 | **0.016** | 1.12 |
| **Kafr El-Zayat** | 0.041 | 3 | 115 | 0.852 | 0.167 |
| **Shubra El-Kheima** | -0.47 | -69 | 259 | **<0.0001** | -2.6 |
| **Badrasheen** | 0.017 | 1 | 93 | 1 | -0.21 |

NB: Slope highlight color is from largest positive (red) to largest negative (blue).

Bold p-value is significant either increasing (red) or decreasing (blue) trends.

Table S2: Homogeneity test (two-tailed) parameters for the significant monotonic trends in the in-situ SO_2_ air pollutant.

|  | **SO_2_** | **Mansoura1** | **Nasr City** | **Beni Suef1** | **Zakazik** | **Al-Salam City** | **Alexandria** | **6th October** | **Qaha** | **Kolaly** | **Suez** | **Mohandeseen** | **Abu Zaabal** | **Giza Square** |
| --- | --- | --- | --- | --- | --- | --- | --- | --- | --- | --- | --- | --- | --- | --- |
| **Pettitt's test:** | **K** | 627 | 641 | 438 | 708 | 518 | 275 | 693 | 888 | 697 | 897 | 757 | 1011 | 439 |
|  | **t** | May-17 | May-16 | Aug-18 | Jun-18 | May-18 | Aug-20 | Sep-17 | Jul-17 | Mar-19 | Sep-18 | May-15 | Oct-18 | Dec-14 |
|  | **p-value** | **< 0.0001** | **0.015** | **0.057** | **< 0.0001** | **0.004** | **0.135** | **< 0.0001** | **0.000** | **< 0.0001** | **< 0.0001** | **< 0.0001** | **< 0.0001** | **0.014** |
|  | **mu1** | 13.46 | 21.96 | 10.94 | 12.3 | 10.52 | 10.24 | 12.44 | 12.34 | 29.16 | 16.32 | 15.3 | 17.5 | 7.22 |
|  | **mu2** | 23.72 | 14.39 |  | 6.87 | 14 |  | 22.65 | 17 | 15.1 | 32.46 | 28.96 | 29 | 17.54 |
| **SNHT test:** | **T0** | 20.497 | 15.06 | 4.23 | 21.62 | 15.118 | 20.82 | 23.43 | 18.24 | 17.08 | 35.67 | 21.51 | 29.91 | 17.50 |
|  | **t** | Jul-18 | May-16 | Aug-18 | Jun-18 | Dec-14 | Sep-16 | Sep-17 | Sep-15 | Nov-19 | Oct-18 | May-15 | Sep-19 | Dec-14 |
|  | **p-value** | **0.000** | **0.010** | **0.412** | **< 0.0001** | **0.001** | **0.002** | **< 0.0001** | **0.000** | **0.001** | **< 0.0001** | **< 0.0001** | **< 0.0001** | **0.000** |
|  | **mu1** | 14.05 | 21.96 | 10.94 | 12.3 | 7.3 | 20 | 12.44 | 9.95 | 28.12 | 16.5 | 15.3 | 18.19 | 7.22 |
|  | **mu2** | 25.52 | 14.39 |  | 6.87 | 12.92 | 9.23 | 22.65 | 16.26 | 12.13 | 32.81 | 28.96 | 34.4 | 17.54 |
| **Buishand's test:** | **Q** | 17.12 | 15.87 | 8.41 | 18.95 | 13.11 | 9.80 | 19.17 | 17.38 | 15.87 | 23.91 | 17.91 | 19.73 | 11.68 |
|  | **t** | May-17 | May-16 | Aug-18 | Jun-18 | May-18 | Sep-16 | Sep-17 | Oct-15 | Mar-19 | Oct-18 | May-15 | Nov-18 | Dec-14 |
|  | **p-value** | **0.000** | **0.000** | **0.181** | **< 0.0001** | **0.003** | **0.022** | **< 0.0001** | **0.000** | **0.000** | **< 0.0001** | **< 0.0001** | **< 0.0001** | **0.013** |
|  | **mu1** | 13.46 | 21.96 | 10.94 | 12.3 | 10.52 | 20 | 12.44 | 10.13 | 29.16 | 16.5 | 15.3 | 17.56 | 7.22 |
|  | **mu2** | 23.72 | 14.39 |  | 6.87 | 14 | 9.23 | 22.65 | 16.3 | 15.1 | 32.81 | 28.96 | 29.32 | 17.54 |

NB: Increasing and decreasing trends are shown in red and blue, respectively.

Table S3: Mann-Kendall monotonic trends and Sen’s slope (alpha=0.05) parameters of the in-situ NO_2_ air pollutant.

| **Station** | Kendall's tau | S' | Var(S') | p-value | **Slope** |
| --- | --- | --- | --- | --- | --- |
| **Mansoura1** | -0.12 | -11 | 153.67 | 0.42 | -1.35 |
| **New Beni Suef** | 0.52 | 23 | 58.33 | **0.004** | 4.16 |
| **Al-Maadi** | -0.114 | -13 | 195 | 0.39 | -0.24 |
| **Masr El-Gadida** | -0.432 | -32 | 114 | **0.004** | -11.08 |
| **Dumiyat** | -0.009 | -1 | 187 | 1 | 0.47 |
| **Al-Salam City** | 0 | 0 | 126 | 1 | -0.74 |
| **6th October** | 0.184 | 14 | 116 | 0.23 | 1.23 |
| **Qaha** | 0.26 | 19 | 107 | 0.08 | 1.57 |
| **Al-Kolaly** | 0 | 0 | 150 | 1 | 0.54 |
| **Mohandeseen** | 0.21 | 15 | 107 | 0.176 | 3.25 |
| **Qasr Ainy** | -0.201 | -30 | 274.67 | 0.08 | -2.33 |
| **Ain Sukhna** | 0.62 | 44 | 116 | **<0.0001** | 9.18 |
| **Abu Zaabal** | 8 | 44 | 0.29 | 0.05 | 1.67 |
| **Helwan University** | 0.09 | 8 | 51.67 | 0.33 | -0.04 |
| **Naser Institute** | -0.41 | -37 | 145.67 | **0.003** | -3.34 |
| **Abbasiya** | 0.08 | 8 | 112.67 | 0.51 | 0.8 |
| **Giza Square** | -0.023 | -2 | 144.67 | 0.934 | 2.37 |
| **Kafr El-Zayat** | -0.248 | -12 | 65.33 | 0.17 | -1.83 |

NB: Slope highlight color is from largest positive (red) to largest negative (blue).

Bold p-value is significant either increasing (red) or decreasing (blue) trends.

Table S4: Homogeneity test (two-tailed) parameters for the significant monotonic trends in the in-situ NO_2_ air pollutant.

|  | **NO_2_** | **New Beni Suef** | **Masr El-Gadida** | **Ain Sukhna** | **Naser Institute** |
| --- | --- | --- | --- | --- | --- |
| **Pettitt's test:** | **K** | 187 | 445 | 489 | 405 |
|  | **t** | Sep-15 | Feb-17 | Aug-16 | Jan-18 |
|  | **p-value** | **0.066** | **< 0.0001** | **< 0.0001** | **0.020** |
|  | **mu1** | **24.28** | **62.52** | **26.23** | **34.15** |
|  | **mu2** |  | **31.45** | **49.08** | **22.29** |
| **SNHT test:** | **T0** | 8.092 | 20.115 | 19.813 | 9.793 |
|  | **t** | Sep-15 | Feb-17 | Aug-16 | Jan-18 |
|  | **p-value** | **0.113** | **< 0.0001** | **< 0.0001** | **0.022** |
|  | **mu1** | **24.28** | **62.52** | **26.23** | **34.15** |
|  | **mu2** |  | **31.45** | **49.08** | **22.29** |
| **Buishand's test:** | **Q** | 6.907 | 15.367 | 16.049 | 12.078 |
|  | **t** | Sep-15 | Feb-17 | Aug-16 | Jan-18 |
|  | **p-value** | **0.102** | **< 0.0001** | **< 0.0001** | **0.008** |
|  | **mu1** | **24.28** | **62.52** | **26.23** | **34.15** |
|  | **mu2** |  | **31.45** | **49.08** | **22.29** |

NB: Increasing and decreasing trends are shown in red and blue, respectively.

Table S5: Mann-Kendall monotonic trends and Sen’s slope (alpha=0.05) parameters of the in-situ PM_10_ air pollutant.

| **Station** | Kendall's tau | S' | Var(S') | p-value | **Slope** |
| --- | --- | --- | --- | --- | --- |
| **Mansoura1** | -0.35 | -35 | 170.33 | **0.009** | -5.375 |
| **Nasr City** | -0.17 | -37 | 424.33 | 0.08 | -4 |
| **Beni Suef1** | -0.01 | -1 | 94.33 | 1 | -0.5 |
| **Zaqaziq** | -0.80 | -79 | 166.33 | **<0.0001** | -19.75 |
| **New Cairo** | 0.08 | 11 | 247.67 | 0.52 | 0.2 |
| **Al-Maadi** | -0.201 | -41 | 406.33 | **0.047** | -7 |
| **Masr El-Gadida** | 0.5 | 58 | 198 | **<0.0001** | 7.81 |
| **Dumiyat** | 0.21 | 35 | 321.67 | 0.06 | 6.57 |
| **Al-Salam City** | 0.40 | 26 | 43 | **0** | 5.31 |
| **6th October** | 0.11 | 14 | 232.67 | 0.39 | 2.6 |
| **Qaha** | -0.35 | -54 | 292 | **0.002** | -6.79 |
| **Al-Kolaly** | -0.39 | -37 | 153.67 | **0.004** | -7.77 |
| **Mohandeseen** | -0.52 | -102 | 392 | **<0.0001** | -10.92 |
| **Qasr Ainy** | 0.02 | 4 | 339.33 | 0.87 | -0.5 |
| **Tebbin** | 0.12 | 24 | 384 | 0.24 | 2.48 |
| **Al-Mehala Al-Kubra** | -0.12 | -11 | 151.67 | 0.42 | -3.69 |
| **Abu Zaabal** | -0.04 | -9 | 374.67 | 0.68 | 0.67 |
| **Shibin Al-Kom** | -0.50 | -65 | 233.67 | **<0.0001** | -12.5 |
| **Helwan University** | 0.04 | 4 | 145.33 | 0.80 | 3.17 |
| **Naser Institute** | -0.24 | -35 | 272.33 | **0.04** | -9.25 |
| **Abbasiya** | 0.05 | 9 | 315.67 | 0.65 | 0.42 |
| **Al-Massara** | -0.24 | -36 | 268.67 | **0.03** | -3.80 |
| **Assut City** | -0.08 | -4 | 64 | 0.71 | -2.92 |
| **Giza Square** | -0.08 | -10 | 180.67 | 0.50 | -3.92 |
| **Kafr El-Zayat** | -0.35 | -48 | 249.33 | **0.003** | -8.47 |
| **Shubra El-Kheima** | -0.45 | -76 | 197 | **<0.0001** | -12.97 |
| **Badrasheen** | -0.03 | -2 | 92.67 | 0.92 | -5.94 |

NB: Slope highlight color is from largest positive (red) to largest negative (blue).

Bold p-value is significant either increasing (red) or decreasing (blue) trends.

Table S6: Homogeneity test (two-tailed) parameters for the significant monotonic trends in the in-situ PM_10_ air pollutant.

|  | **PM10** | **Mansoura1** | **Zaqaziq** | **Maadi** | **Masr El-Gadida** | **Al-Salam City** | **Qaha** | **Al-Kolaly** | **Mohandeseen** | **Shibin Al-Kom** | **Naser Institute** | **Al-Massara** | **Kafr El-Zayat** | **Shubra El-Kheima** |
| --- | --- | --- | --- | --- | --- | --- | --- | --- | --- | --- | --- | --- | --- | --- |
| **Pettitt's test:** | **K** | 447 | 717 | 1134 | 397 | 307 | 618 | 434 | 905 | 718.000 | 807.000 | 435.000 | 547.000 | 1099.000 |
|  | **t** | Jan-17 | May-18 | May-16 | Dec-15 | Aug-15 | May-18 | Feb-15 | Sep-15 | Jul-17 | Nov-18 | Feb-19 | Nov-16 | Dec-16 |
|  | **p-value** | **0.011** | **< 0.0001** | **< 0.0001** | **0.125** | **0.057** | **0.004** | **0.011** | **< 0.0001** | **0.000** | **< 0.0001** | **0.039** | **0.001** | **< 0.0001** |
|  | **mu1** |  | 163.19 | 162.75 | 180.59 | 145.81 | 142.42 | 235.81 | 189.13 | 161.3 | 144.87 | 183.87 | 165.93 | 181.96 |
|  | **mu2** |  | 100.17 | 114.38 |  |  | 111.28 | 166.84 | 130.96 | 100.88 | 98.43 | 120.9 | 131.7 | 104.34 |
| **SNHT test:** | **T0** | 20.859 | 31.815 | 23.875 | 8.014 | 6.475 | 11.740 | 23.068 | 30.331 | 23.370 | 18.974 | 13.968 | 12.399 | 34.611 |
|  | **t** | Feb-15 | Feb-15 | May-16 | Dec-15 | Sep-14 | May-18 | Aug-14 | Feb-15 | Sep-15 | Jan-19 | Feb-19 | Nov-16 | Dec-16 |
|  | p-value | **0.000** | **0.000** | **0.000** | **0.097** | **0.190** | **0.009** | **< 0.0001** | **< 0.0001** | **0.002** | **0.000** | **0.003** | **0.008** | **< 0.0001** |
|  | **mu1** | 141.11 | 234.55 | 162.75 | 180.59 | 145.81 | 142.42 | 269.5 | 198.7 | 186.16 | 144.12 | 183.87 | 165.93 | 181.96 |
|  | **mu2** | 89.32 | 121.36 | 114.38 |  |  | 111.28 | 168.38 | 134.23 | 108.83 | 95.81 | 120.9 | 131.7 | 104.34 |
| **Buishand's test:** | **Q** | 13.091 | 17.619 | 21.956 | 17.504 | 8.511 | 13.939 | 13.982 | 21.913 | 17.918 | 17.535 | 10.956 | 14.042 | 25.085 |
|  | **t** | May-16 | Jan-17 | May-16 | Jul-17 | Aug-15 | May-18 | Aug-14 | Sep-15 | Sep-15 | Jul-18 | Feb-19 | Nov-16 | Dec-16 |
|  | p-value | **0.002** | **< 0.0001** | **< 0.0001** | **0.001** | **0.083** | **0.005** | **0.001** | **< 0.0001** | **< 0.0001** | **< 0.0001** | **0.034** | **0.002** | **< 0.0001** |
|  | **mu1** | 114.69 | 182.63 | 162.75 | 180.59 | 145.81 | 142.42 | 269.5 | 189.13 | 186.16 | 146.33 | 183.87 | 165.93 | 181.96 |
|  | **mu2** | 86.23 | 112.46 | 114.38 |  |  | 111.28 | 168.38 | 130.96 | 108.83 | 101.21 | 120.9 | 131.7 | 104.34 |

NB: Increasing and decreasing trends are shown in red and blue, respectively.
